# Association between transfer for surgery and mortality and disability among neonates in high income countries—A systematic review with meta-analysis

**DOI:** 10.1371/journal.pone.0327971

**Published:** 2025-07-31

**Authors:** Haribalakrishna Balasubramanian, Abhishek Srinivas, Prathamesh Khedkar, Anitha Ananthan, Diwakar Mohan, Nandkishore Kabra, Sanjay Patole

**Affiliations:** 1 Department of Neonatology, Surya Hospitals, Mumbai, India; 2 Department of Neonatology, Seth GS Medical College and King Edward Memorial Hospital, Mumbai, India; 3 Department of International Health, John Hopkins Bloomberg School of Public Health, Baltimore, Maryland, United States of America; 4 Neonatal Directorate, King Edward Memorial Hospital for Women, Subiaco, Western Australia, Australia; 5 University of Western Australia, School of Medicine, Crawley, Western Australia, Australia; St Paul's Hospital Millennium Medical College, ETHIOPIA

## Abstract

Birthing in a hospital with on-site surgical facilities (co-located care) is considered preferable for neonates with surgical conditions. However, it may not always be feasible. Whether transfer of surgical neonates from birth hospital to a surgical facility affects the outcomes of this cohort is unclear. We conducted a systematic review to investigate the association of birth location/transfer with all-cause mortality and disability among neonates with congenital/acquired surgical conditions. Data Sources from PubMed, Embase, CINAHL, and Web of Science were searched till December 2024. Studies from high-income countries (HICs) comparing infants transferred to a surgical center for surgery versus infants born and operated in a co-located care facility were included. Random effects model was used for meta-analysis. The quality of studies and certainty of evidence were assessed using Newcastle-Ottawa Scale and the GRADE framework respectively. The primary outcomes of interest were all-cause mortality and neurodevelopmental impairment at latest follow up. A total of 61 studies from 20 HICs were included. Compared to co-located care, transfer for surgery did not increase the odds of risk-adjusted and crude mortality in neonates with congenital diaphragmatic hernia [adjusted odds ratio (aOR):0.86 (0.49 to 1.49), 5 studies, 8366 infants; crude OR:0.68 (0.51 to 0.91, i.e., decreased mortality), 22 studies, 12970 infants], critical congenital heart disease [aOR:0.79 (0.42 to 1.48), 3 studies, 13485 infants; OR:1.04 (0.66 to 1.64), 10 studies, 14447 infants], surgical necrotizing enterocolitis [aOR:0.99 (0.61 to 1.61), 4 studies, 5891 infants; OR:1.03 (0.64 to 1.65), 5 studies, 5915 infants], gastroschisis [aOR:1.07 (0.68 to 1.68), 2 studies, 5294 infants; OR:0.80 (0.48 to 1.35), 11 studies, 8708 infants], tracheo-oesophageal fistula [aOR:0.97 (0.39 to 2.39), 1 study, 937 infants; OR:0.62 (0.37 to 1.04), 4 studies, 4050 infants], congenital or perinatal intestinal conditions [OR:2.69 (0.26 to 28.34), 4 studies, 1799 infants]. Neurodevelopmental outcomes between the groups were comparable in the three studies that reported this outcome. Whilst many included studies were of good quality, certainty of evidence was very low due to their observational design and heterogeneity. In conclusion, transfer of neonates from the birth hospital to another facility for surgical intervention was not associated with increased risk of mortality or disability. The evidence from this comprehensive meta-analysis would be useful for clinicians, parents and health policy makers.

**Systematic review registration:** PROSPERO CRD 42024565651.

## Introduction

Infants requiring surgical intervention during the neonatal period are at higher risk of mortality and morbidities. Whilst birthing in a hospital with on-site surgical facilities (co-located care) is considered preferable for such infants to optimize care, it may not always be feasible. Nearly 40–45% of neonates with major surgical conditions need transfer from the birth location to a surgical facility for specialist care [1,2]. Unlike very preterm neonates without surgical conditions [3–5], the association of birth location with adverse outcomes is not clear among neonates with various surgical conditions. A systematic review that covered gastroschisis, tracheoesophageal fistula (TEF) and congenital diaphragmatic hernia (CDH) reported that the evidence in this area is conflicting [6].

Granger et al. reported outcomes after surgically managed necrotising enterocolitis (NEC) and focal intestinal perforation (FIP) in infants <32 weeks requiring transfer to or presenting in a single surgical center. Being transferred was associated with increased all-cause mortality and mortality attributable to NEC or FIP, but no differences in neurodevelopmental outcomes among survivors [7]. Other investigators report that survival and early postoperative mortality was not affected by the distance from hospital of birth to a surgical center in neonates with critical cardiac conditions including hypoplastic left heart syndrome (HLHS) and transposition of great arteries (TGA) [2,8].

Considering that surgical neonates account for 20–60% of emergency neonatal transports [9,10], a comprehensive evaluation of outcomes associated with neonatal surgical transfers is required to guide perinatal care including triaging and neonatal retrieval priorities in this high-risk cohort. Apart from the severity of the underlying condition and retrieval distance, the standard of neonatal care at the hospital of birth, and importantly, during transport is critical for survival of neonates with major surgical conditions. Hospital mortality in this cohort varies from 4–40% in high income countries (HICs) [11,12] with better survival in centers with high operative case volumes [13,14]. Given the significance of the underlying issues, we aimed to systematically assess if transfer from the birth hospital to a surgical center is associated with mortality and disability among neonates with major surgical conditions in HICs.

## Methods

We followed the Cochrane handbook of systematic reviews for observational studies [15] for conducting and the MOOSE (Meta-analyses Of Observational Studies in Epidemiology) guidelines for reporting this systematic review [16]. The protocol was registered in the international prospective register of systematic reviews (PROSPERO id: CRD42024565651).

### Type of studies

Prospective or retrospective non-randomised (e.g., Cohort, case-control) studies conducted in HICs (World Bank classification 2024) [17] were included. We excluded studies conducted in low- and middle-income countries since protocols for regionalisation of care for surgical neonates may not be uniformly well established in such countries. Randomized controlled trials (RCTs) were eligible for inclusion but not expected for obvious reasons.

### Search strategy

We searched PubMed, Embase, CINAHL and Web of science, Google scholar, and conference abstracts from conception till December 2024. Reference lists of included studies and related systematic and narrative reviews were searched to identify additional or ongoing studies. No language restrictions were applied. The search strategy is included in the supplement. (S1 File)

### Inclusion criteria

We included studies that evaluated the impact of hospital transfer on neonates with major surgical conditions. We compared two groups of infants: (1) those transferred from the birth hospital to a surgical center (surgical transfer group); (2) infants born and operated in a hospital with surgical facility (co-located care group). We included infants that underwent open surgical procedures, minimal access surgeries or catheter-based interventions. Infants were categorized irrespective of the time of diagnosis (antenatal/postnatal) or presentation (congenital/ acquired) of the surgical condition.

Infants who underwent surgery beyond the neonatal period (>28 days) were included only if they were still in the hospital since birth when they underwent surgery.

### Exclusion criteria

Elective inguinal hernia repairs and retinal laser photoablations were excluded. When two or more studies selected infants from the same hospital or database, with overlapping study time frames, we selected the largest and the most recent study reporting the desired outcomes for our analyses.

### Surgical conditions

Our prespecified list of major congenital or acquired surgical conditions included:

(1) Gastrointestinal: (a) *Congenital*: Esophageal atresia (EA) with or without TEF, congenital diaphragmatic hernia (CDH), gastroschisis, exomphalos, intestinal atresia, meconium ileus/peritonitis, intestinal malrotation/volvulus, Hirschsprung disease, anorectal malformations. (b) *Acquired*: NEC, spontaneous intestinal perforation.
(2) Cardiovascular: Critical congenital heart defects (CCHD) including TGA, HLHS, hypoplastic right heart syndrome, truncus arteriosus and other ductal dependent cardiac lesions
(3) Neurological: Neural tube defects, congenital or acquired hydrocephalus
(4) Others: Congenital pulmonary and airway malformations, urogenital malformations.

Outcomes:

The primary outcome for this systematic review was all-cause mortality (mortality during primary hospitalization or at latest follow up). Infants that died at the surgical center before surgery and infants with surgical diagnoses managed conservatively were included in outcome evaluation. Secondary outcome was long-term neurodevelopment including motor, language or cognitive impairment (assessed using validated neurodevelopmental tools), cerebral palsy, visual and hearing impairment, and autism.

### Differences between the PROSPERO protocol and the final review

We stated in our protocol that infants operated beyond the neonatal period will be excluded, whereas in our final review, infants who underwent surgery beyond the neonatal period (>28 days) were included if they were still in the hospital since birth when they underwent surgery. We also prespecified that inguinal hernia cases will be excluded. In the actual review, although elective surgeries of uncomplicated inguinal hernias were excluded, inguinal hernias with complications such as obstruction, gangrene and incarceration that need emergency surgery were planned to be included.

### Study selection and data extraction

Two authors independently screened studies to assess eligibility for inclusion in our review. Discrepancies in study selection were resolved in consensus with the third author. Authors (AS and PK) independently extracted information on prespecified outcomes of mortality and developmental disability and assessed the risk of bias (ROB) from the included studies. We extracted the following characteristics for each surgical condition: country, data source, study design and timeframe, proportion of infants with antenatal diagnosis and serious congenital/chromosomal anomalies, age at hospital transfer and surgery, definition of co-located care and surgical transfer. We collected information on pre and post-surgical mortality, pre-transfer mortality, palliative care, non-surgical management rates, and need for extracorporeal membrane oxygenation.

### Statistical analysis

Meta-analysis was performed using the random effects model. Effect sizes were reported as odds ratios (OR) and 95% confidence intervals (CI). For studies reporting risk adjusted mortality, ORs and standard errors were logarithmically transformed and pooled using the generic inverse variance method. The I^2^ statistic was used to assess statistical heterogeneity. I^2^ values of 25% or less, 25% to 75%, and >75% were considered as having low, moderate, and high heterogeneity, respectively. The quality of included observational studies was assessed using the Newcastle-Ottawa Scale [18]. Publication bias was assessed using funnel plots if there were at least 10 included studies for a given surgical condition. We planned analysis of mortality by subgroups based on gestational age and severity of the surgical condition. Statistical significance was set at p < 0.05 and all tests were two-tailed. Revman Version 5.4 was used for all analyses. Certainty of evidence was assessed using GRADE recommendations [19].

## Results

**Fig 1:**
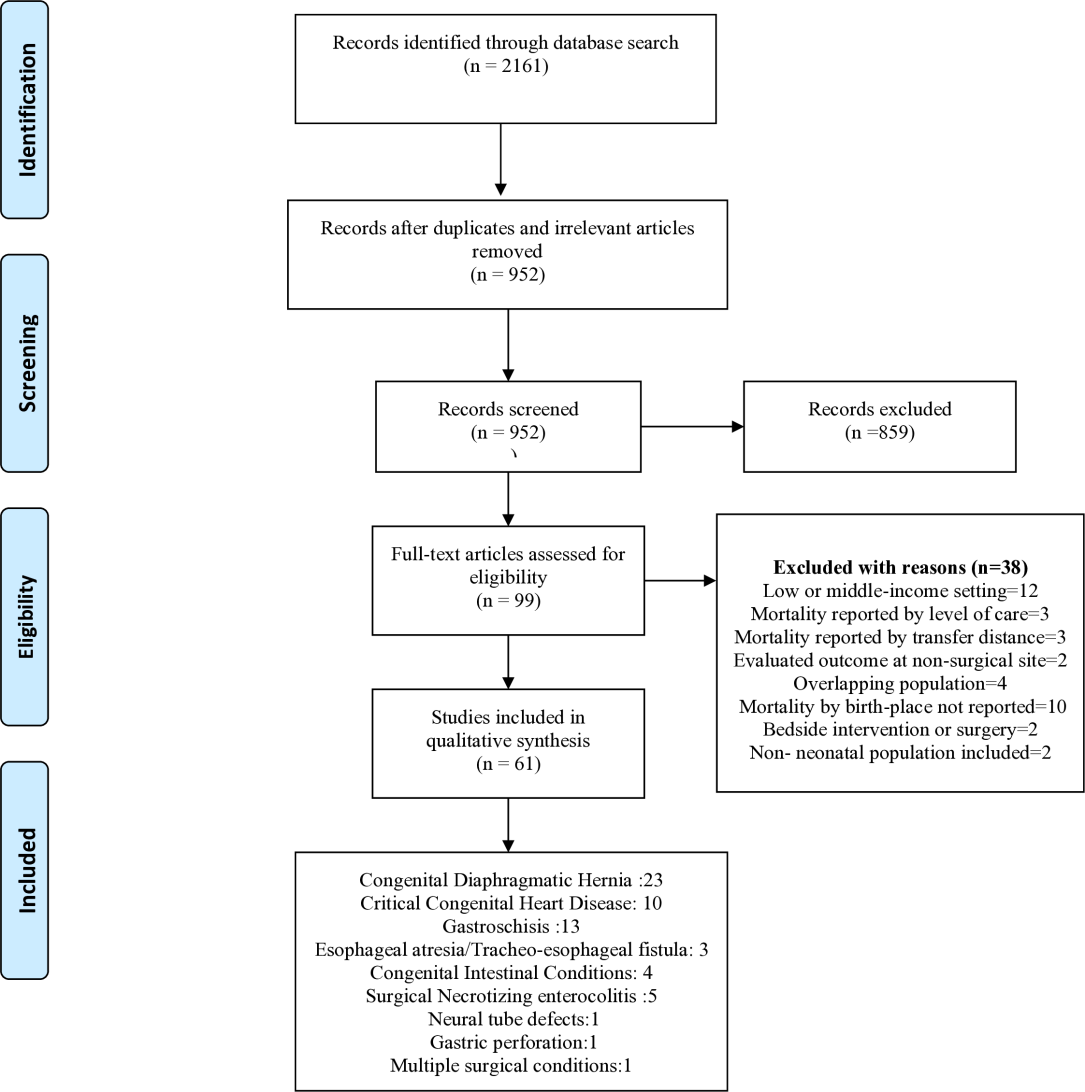
PRISMA flow diagram for selection of studies.

Our literature search yielded 2161 citations. After initial eligibility assessment, 99 articles were read in full text and a total of 61 studies from HICs reporting the effect of hospital transfers for surgical intervention were included (Fig 1).

The sample sizes of the included studies varied from 13 to 12,106 surgical infants. Algert 2008 evaluated outcomes separately for CDH, gastroschisis, exomphalos, TGA, esophageal atresia (EA) and neural tube defects with hydrocephalus [20]. Three studies reported long-term neurodevelopmental outcomes.

Four studies (Gastroschisis: 2, CDH:1, TGA:1) evaluated mortality but did not report point estimates for conducting meta-analysis. Included studies abstracted data from hospital medical records, population-level inpatient databases, disease registries or health provider surveys. The characteristics of included studies are summarized in Table 1 and additional study characteristics are shown in S1 and S3 Table. Characteristics of excluded studies were tabulated in S2Table.

**Table 1:** Characteristics of included studies.

Study ID, Country	Data sources	Years	Inclusion criteria	Proportion of infants with prenatal diagnosis	Exclusion criteria	Chromosomal/serious congenital anomalies	Total Sample size (Co-located/Transfer)	Time to surgery	Primary outcome
**Congenital Diaphragmatic Hernia (CDH)**									
Algert 2008, Australia [20]	New South Wales hospitals inpatient database	2001-2003	Livebirths with one of the selected defects- gastroschisis, exomphalos, diaphragmatic hernia (CDH), esophageal atresia, transposition of great arteries (TGA), spina bifida with hydrocephalus.		Infants with multiple lethal or uncorrectable defects or unconfirmed diagnoses, and infants diagnosed outside of the neonatal period		287 (163/124)		Mortality at 1 year
Al- Shanafey 2002, Canada [21]	IWK Grace Health centre, Halifax, Nova Scotia	1973-1999	Live newborns with bochdalek type CDH with respiratory distress within 4 hours of life	14/81 total infants	Prenatal death, and death immediately after delivery, lethal cardiac anomalies	Excluded	81 (40/41)		Death before discharge
Al Shareef 2024, Saudi Arabia [22]	Best care health software and physical records of hospital admissions at King Abdelaziz Medical City	2000-2021	All live births with CDH regardless of timing of diagnosis, type, size, site	26/45 total infants		5/45 total infants	45 (36/9)		Death before discharge
Aly 2010, USA [23]	De-identified data from national agency for health care research and quality and the KIDS’ database	1997 - 2004	CDH infants admitted within 8 days of age, operated within the first 6 weeks of life and/or died before the surgery		Brain anomalies, abdominal wall defects, multiple congenital anomalies and chromosomal disorders	Excluded	2140 (1120/1020)		Death before discharge
Boloker 2002, USA [24]	Records from Children hospital of New York	1992-2000	All CDH neonates irrespective of associated anomalies, degree of pulmonary hypoplasia and medical condition at birth	65/67 (co-located group) 25/53 (transfer group)	Patients transferred postoperatively	17/67 (co-located group) 6/53 (transfer group)	120 (67/53)	98 hours (co-located group) 118 hours (transfer group)	Death before discharge
Bryner 2009 USA [25]	CDH study group registry data from more than 50 centres around the world	1995- 2005	All patients who underwent repair of CDH while or after ECMO therapy		Less severe cases in which ECMO was not required (2830 cases) and very severe cases of CDH in which all patients died before repair (204 cases)		636 (193/443)		Death before discharge
Carmichael 2020, USA [26]	California Perinatal Quality Care Collaborative (CPQCC)	2006-2011	Infants with CDH whose birth certificates were linked to hospital discharge records		Infants whose birth certificates were not linked to hospital discharge records, those who had fetal surgery, and records with potential data errors	80/577 total infants	577 (220/357)		Death during infancy
Dahlheim 2003, Germany [27]	University Children’s hospital, Mannheim	December 1997- June 2001	Neonates with CDH treated between 1997 and 2001	All co-located group infants, 6/26 transfer group infants			50 (24/ 26)		In-hospital mortality
Finer 1998, Canada and USA [28]	Hospital records Royal Alexandra hospital	February 1989 – August 1996	Near-term (>34 weeks’ gestation) newborns with CDH	20/23 co-located group infants			65 (23/42)		Death before discharge
Gallot 2007, France [29]	Central-Eastern France Registry data	1986-2003	Central Eastern France registry infants aged 1 year or younger with a verified diagnosis of CDH.	167/387 total infants		186/501 total infants	387 (176/211)		Death during neonatal period
Goldshore 2023, USA [30]	Pulmonary Hypoplasia Program cohort (PHP), data at Children’s Hospital of Philadelphia	February 2006- March 2021	Prenatally diagnosed isolated CDH who received any prenatal care at a single fetal centre		Intrauterine fetal demise or withdrew from participation.		418 (322/96)	Median of 10 days	Death at discharge
Khachane 2021, Australia [31]	Hospital medical records and neonatal database of Children’s Hospital of Westmead.	2002-2018	All postnatal infants with CDH including those who died in the perinatal NICU prior to transfer	118/120 (co-located group) 1/39 (transfer group)	In- utero deaths	21/159 total infants	159 (120/39)		In hospital mortality
Kim 2009, Michigan, USA [32]	Medical records of CS Mott Children’s Hospital	2004-2008	Full term neonates who underwent thoracoscopic CDH repair	6/15 total infants			15 (5/10)	Mean 5.7 days	Deaths before discharge
Maldonado 2024, UK [10]	UK National Neonatal research database	2012-2020	All neonates with diagnosis of CDH		Infants with inconsistent transfer patterns and those with multiple congenital (non-cardiac) surgical abnormalities	excluded	997 (660/337)		Death before discharge
Nagata 2013, Japan [33]	Questionnaire based survey from 109 hospitals/ perinatal centres affiliated with the Japanese Society of Paediatric Surgeons	2006-2010	Neonates diagnosed with CDH	442/614 total infants	CDH diagnosed after 28 days of age	95/614 total infants	614 (449/165)	61 hours (30–99 hours)	Death before discharge
Nakayama 1985, California [34]	Clinical records of University of California, San Francisco	July 1979 – June 1982	Liveborn infants with CDH	3/7 co- located group infants		3/17 total infants	17 (7/10)	<24 hours of age	Death before discharge
Nasr 2011, Canada [35]	Canadian Paediatric Surgery Network (CAPSNet) covering 16 tertiary perinatal centres in Canada	2005-2008	Infants with antenatally diagnosed CDH	All infants	Postnatally diagnosed CDH		140 (75/65)	Median of 5 days (co-located group) and 6.5 days (transfer group)	Death before discharge
Peiffer 2024, USA [36]	Texas Hospital Inpatient Discharge Public Use Data File (PUDF), a state-wide hospital discharge database	2013-2021	Neonates and infants with CDH who were discharged before one year of age		Neonates transferred outside of Texas children’s hospital		1314 (656/658)		In hospital mortality
Reyes 1998, California [37]	Medical records of University of California - Irvine Medical Centre (UCIMC)	1993-1996	All neonates with CDH	6/8 (co-located group) 3/14 (transfer group)	Infants that had fetal surgery or repair prior to transfer to UCIMC	2/22 total infants	22 (8/14)		Death before discharge
Rocha 2008, Portugal [38]	Hospital de São João neonatal intensive care unit (NICU) database	1997-2006	All neonates diagnosed with CDH	42/61 total infants	Neonates with diaphragmatic eventration	5/61 total infants	61 (46/15)	Median of 4 days	In hospital mortality
Sola 2010, USA [39]	Kids’ inpatient database of USA	1997,2000, 2003,2006	Neonates with CDH and < 8 days of age		Missing data, transferred out of surgical centre		2571 (1680/891)		Death at discharge
Stopenski 2021, USA [40]	Congenital diaphragmatic hernia study group (CDHSG) database of 80 international centres	2006-2019	Prenatally diagnosed CDH	All infants	Postnatally diagnosed CDH, bilateral diaphragmatic defects	8.9% of co-located group 8.1% of transfer group	4195 (3087/1108)		In-hospital mortality
Teo 2020, Singapore [41]	Hospital records of KK Women’s and Children’s hospital	January 2002 and June 2005	Infants with antenatally and postnatally diagnosed CDH			1/19 infants	19 (17/2)	Median of 5 days	Death before discharge
Wynn 2013, USA [42]	DHREAMS (collaborative study involving 7 US universities- Columbia, Washington, Vanderbilt University, University of Cincinnati, Pittsburgh, Nebraska and Michigan).	2005-2010	Radiologically confirmed CDH and born or transferred to the participating centre within first week of life	76.4% total infants		38% of total infants	220 (140/80)	Mean: 8 days	Mortality at discharge, at 14 days of life and 60 days of life
**Critical Congenital Heart Diseases (CCHD)**									
Bennett 2010, Washington [43]	Washington State Comprehensive Hospital Abstract Reporting System (CHARS) of all inpatient hospitalization	1987–2006	Infants with ductal-dependent cardiac anomalies that underwent cardiac procedure (interventional or surgical, palliative or corrective) within 30 days of birth.		No cardiac procedure within 1 year, first procedure at >30days of life, differential diagnosis at first procedure	7/285 (co-located group) 8/538 (transfer group)	823 (285/538)	< 30 days	Mortality within 90 days after birth
Cave 2023, UK [44]	Local database of all fetal cardiac diagnoses at Leeds Children Hospital, UK.	2015-2020	Antenatally diagnosed simple transposition of the great arteries (TGA)	All infants	Additional cardiac diagnosis other than ventricular septal defect or coarctation of the aorta. All postnatally diagnosed TGA during study period.	Excluded	54 (51/3)		Mortality at 90 days
Cloete 2018, New Zealand [45]	National Paediatric Cardiology and Cardiac Surgical databases and New Zealand Ministry of Health data, New Zealand perinatal and maternal mortality review committee data	2006-2014	All infants with d-TGA and critical aortic arch obstruction requiring intervention within the first 28 days	97/253 total infants	Chromosomal anomalies or major non cardiac anomaly or preterm <35 weeks of gestation	Excluded	253 (106/147)	<28 days	Mortality in the first 28 days
Garne 2007, Europe [46]	Central EUROCAT database-population based registry of 7 European countries (Belgium, UK, Netherlands, Spain, Germany, Denmark, Switzerland)	1996-2000	Cases with TGA and ventricular septal defect (VSD)	8% of total infants	associated malformations (cardiac or noncardiac, syndromes or karyotype anomalies)	Excluded	93 (13/80)		Deaths by 1 year of age
Hamzah 2020, USA [47]	Healthcare Cost and Utilization Project (HCUP). National Inpatient Sample (NIS) database of hospitals from 47 US states	2002–2016	All neonates who were ≤ 28 days of age at the time of admission and diagnosed with Hypoplastic Left Heart Syndrome (HLHS).		Patients with total anomalous pulmonary venous return (TAPVC)	Excluded TAPVC (2227 out of 18866)	12106 (7904/4202)		In-hospital mortality
Mattia 2024, USA [48]	Society of Thoracic Surgeons and Phoenix Children’s Fetal Cardiology databases	2013-2022	Infants with d-TGA and hypoxia (SpO2 < 70%) who required balloon atrial septostomy within the first 48 h of life.	41/68 total infants	–	–	41 (17/24)	Time to BAS = 1.1 vs 4.5 hours	Mortality before discharge
Purkey 2021, California [49]	California Perinatal Quality Care Collaborative (CPQCC) and the Office of Statewide Health Planning and Development (OSHPD)	2006-2011	Neonates with HLHS		Cases not linked to OSHPD data, fetal surgery, data errors	33/556 total infants	556 (187/369)		Mortality by 28 days Mortality by 1 year
Swartz 2017, USA [50]	New York State Cardiac Surgical database.	2005-2014	Neonates (age < 30 days) who underwent cardiac surgery at the University of Rochester Medical Centre	104/113 (co-located group) 77/268 (transfer group)	Babies discharged from their birth hospital and re-presented within the neonatal period for surgery; premature neonates requiring patent ductus arteriosus ligation; and neonates transferred less than 10 miles		381 (113/268)	Median of 5 days (co-located group) and 8 days (transfer group)	Thirty-day survival
Thomas 2023, USA [51]	Internal cardiac catheterization database and Electronic Medical Record Search Engine (EMERSE) at CS Mott Children’s Hospital, Ann Arbor.	2010- 2021	Neonates with d-TGA or d-TGA physiology who underwent urgent or emergent Balloon atrial septostomy (BAS) at study centre	66/67 (co-located group) 3/29 (transfer group)	Patients who underwent elective BAS at more than 48 h of life, did not undergo a BAS, or had a BAS at an outside institution prior to transfer to study centre.	13/67 (co-located group) 5/29 (transfer group)	96 (67/29)		Mortality within 6 months of life and first year of life
Veal 2019, UK [52]	Local Paediatric Intensive Care Audit Network database and the local specialist paediatric transport team database of South-West England and South Wales	April 2012 – March 2018	Infants diagnosed with TGA requiring arterial switch operation as a definitive procedure and who were born in the South-West of England and South Wales	25/45 total infants			41 (26/15)	Median days for arterial switch: 8 days (Co-located group) 10 days (transfer group)	Mortality before discharge
**Necrotising Enterocolitis (NEC) and Focal Intestinal Perforation (FIP)**									
Fullerton 2016, Boston [53]	Vermont Oxford Network (VON)	January, 2009 to December, 2013	Infants who underwent surgery for NEC		Patients with length of stay less than72 hours, who had NEC but did not have surgery, had surgery for NEC performed at multiple hospitals, had a diagnosis of NEC made at multiple hospitals, transferred between more than two hospitals, or had major congenital anomalies. Infants born, diagnosed or operated at a center other than the reporting center	excluded	4328 (2882/1446)	–	Mortality before discharge
Granger 2022, UK [7]	Transport service database, electronic case records linked with the Northern Neonatal Network of 12 UK neonatal units	2013-2020	Infants with possible NEC or perforation with birth gestation <32weeks		Pan-NEC	2/113 Co-located group 1/92 transfer group	205 (113/92)	–	Mortality attributed to NEC or FIP Two-year development
Kelley-Quon 2012, California [54]	California Patient Discharge Linked Birth Cohort Database	1999–2007	Very low birth weight infants (VLBW) who underwent NEC surgery		Infants with surgery for NEC < 7 days after birth	24/1272 total infants	1272 (866/406)	Mean age of 28 days	Mortality at one year of age
Loh 2001, Australia [55]	Hospital records and NSW NICU study database of 7 perinatal centres in NSW	1992- 1997	Infants of less than 29 weeks gestation with surgical NEC		Infants with no radiological evidence of NEC		124 (52/72)	–	Death before discharge
Zamrik 2018, Germany [56]	Hospital records from Childrens’ Hospital, Oberhausen	2009-2014	VLBW newborns with a birthweight < 1250g having NEC				24 (6/18)	–	Death before discharge, Two-year development
**Gastroschisis**									
Abdel-Latif 2008, Australia [57]	Australian and New Zealand Neonatal Network (ANZNN) of 28 level III NICUs and 16 level II nurseries.	1997-2005	Consecutively admitted infants of gastroschisis in ANZNN nurseries between 1997 and 2005	–	Anterior abdominal wall malformation (exomphalos, prune -belly syndrome), liveborn gastroschisis who died before or during retrieval	73/631 total infants	631 (563/68)	–	Death before discharge
Clark 2010, US [58]	Pediatrix Medical Group data warehouse of 284 US hospitals	1997-2008	Neonates with gastroschisis admitted to NICU	–	Neonates who died in the delivery room or those not admitted to the NICU. Duplicate entries from transfer between consortium units	231/2749 total infants	2749 (2429/320)	–	Death before discharge
Dalton 2016, US [59]	Children’s Mercy Hospital, Kansas City	2010-2015	Infants with gastroschisis who had all stages of the abdominal closure performed at study centre	45/45 (co-located group) 16/18 (transfer group)	complicated gastroschisis (those having an atresia or requiring immediate intestinal resection owing to volvulus or perforation)	–	63 (45/18)	73 hours (co-located group) 79 hours (transfer group)	Death before discharge
Hong 2018, US [60]	Vermont Oxford Network (VON) Expanded Database of 159 US NICUs	2009-2015	Infants with gastroschisis admitted within 28 days and underwent repair or died without undergoing repair at a reporting centre	–	Infants transferred after undergoing gastroschisis repair	1.8% of co-located group 2.3% of transfer group	4663 (3529/1134)		Death before discharge
Kandasamy 2010, Australia [61]	Hospital records from the Townsville hospital, Queensland.	1988-2007	All cases of gastroschisis treated at Townsville hospital	40/40 (co-located group) 5/10 transfer group			50 (40/10)		Death before discharge
Lee 2024, UK [62]	Badgernet electronic medical record system and medical records of King’s College hospital	2008-2017	Antenatally diagnosed gastroschisis	–	Infants that underwent primary closure without preformed silo, missing data records	–	77 (61/16)		In-hospital mortality
Nasr 2012, Canada [63]	Canadian Paediatric Surgery Network	2005-2008	Antenatally diagnosed gastroschisis	All infants		–	395 (237/158)	Median: 2.4 days (co-located group) vs 4.2 days (transfer group)	Death before discharge
Quirk 1996, California [64]	Medical records of Arkansas Children Hospital	June 1987- April 1995	Neonates with gastroschisis	31/32 (co-located group) 7/24 (transfer group)			56 (32/24)	Mean: 3.4 hours (co-located group) vs 6.6 hours (transfer group)	Death before discharge
Rinehart 1999, Mississippi [65]	Hospital data of University of Mississippi Medical Centre	September 1992- June 1998	Infants with diagnosis of gastroschisis or abdominal wall defects	15/23 (co-located group) 0/9 (transfer group)	Infants with other abdominal wall defects (such as omphalocele, cloacal exstrophy, and prune-belly syndrome)		32 (23/9)	<24hours	Death before discharge
Savoie 2014, US [66]	Paediatric Surgery Research Collaborative of 6 US Universities	2008-2013	Patients treated for gastroschisis	95% of co-located group 87% of transfer group	Anterior abdominal wall defects, delay in transport to the treating facility greater than 48h from birth	8% of co-located group 18% of transfer group	528 (286/242)	3.7 days (Co-located group) 5.4 days (transfer group)	30-Day Mortality
Stoodley 1993, UK [67]	Patient records from Bristol Hospital	1981-1990.	Newborns with gastroschisis	17/19 (co-located group) 4/30 (transfer group)		Simple Gastroschisis = 15/19 (co-located group) 25/30 (transfer group)	49 (19/30)	Mean: 3 hours (co-located group) vs 6 hours (transfer group)	Death before discharge
Stringer 1991, London [68]	Case records of Hospital for Sick children and University College hospital, London	13-year period	Newborns with gastroschisis	9/9 (co-located group) 12/31 (transfer group)		13/40 total infants	40 (9/31)	Mean: 4.6 hours (co-located group) vs 6.7 hours (transfer group)	Death before discharge
Youssef 2016, Canada [69]	(CAPSNet) database of Canadian hospitals	2005-2013	All registered gastroschisis patients		Infants with unrelated procedure codes, missing data on gestational age or birth weight	6/695 total infants	Canada: 695 (536/159)		In-patient mortality
**Esophageal Atresia (EA), Tracheo-esophageal Fistula (TEF)**									
Schlee 2022, Germany [70]	Medical, surgical and radiological records of Goethe University hospital, Frankfurt	2009-2019	Infants with EA treated between 2009 and 2019	1/17 (co-located group) 1/35 (transfer group)	Preoperatively suspected long-gap atresia	13/17 (co-located group) 26/35 (transfer group)	52 (17/35)	Median 1 day in each study group	Death by one year of age
Sfeir 2021, France [71]	National Plan for Rare Diseases, population-based registry of 39 French hospitals	2008-2014	Children with lower esophageal fistula	132/991 total infants		535/1007 infants	937 (347/590)	87% infants operated in first 48 hours	3-month mortality
Wang 2014, Florida [72]	KID’s Inpatient Database for children in United States	1997-2009	Neonates with diagnosis of EA/TEF	–	Readmissions and reoperations, missing dates of operation, conflicts in diagnosis, treatment received and discharge status	unclear	3019 (1850/ 1169)	unclear	Mortality before discharge
**Meconium Peritonitis and Intestinal atresia**									
Chen 2019, Taiwan [73]	Hospital records of three Mackay Memorial hospital health systems	2005-2016	Neonates with a diagnosis of perinatal bowel perforation characteristic of meconium peritonitis	17/22 (co-located group) 7/15 (transfer group)	Neonates diagnosed with NEC or focal intestinal perforation		37 (22/15)	30/37 (surgical intervention before 3 days)	Death before discharge
Erickson 2016, USA [74]	The Kids’ Inpatient Database of the US	2012	Patients less than one year old with a principal diagnosis of small intestinal atresia and an associated surgical intervention				1672 (920/752)	Median 2 days (co-located group) vs 1 day (transfer group)	Death before discharge
Paradiso 2011, Italy [75]	Hospital medical records of San Camillo Forlanini hospital	1992-2010	All neonates admitted with meconium related ileus	None	Infants with cystic fibrosis and Hirschsprung’s disease		55 (13/42)		In-hospital mortality
Wong 2021, Hong Kong [76]	Hospital records of Queen Mary Hospital	1997-2019	All neonates with meconium peritonitis	27/35 total infants		1/35 total infants	35 (19/16)	Median: 1 day (co-located group) vs 4 days (transfer group)	Death before discharge
**Others**									
Kancherla 2021, California [77]	California Perinatal Quality Care Collaborative of 139 NICUs	2006-2011	Infants born with spina bifida reported in CPQCC			17 of total infants	603 (288/315)		Mortality within first year of life
Yang 2015, Taiwan [78]	Chang Gung Memorial Hospital database	1997-2008	Neonates with a diagnosis of gastric perforation			3/4 (co-located group) 1/9 (transfer group)	13 (4/9)		Death before discharge

ANZNN, Australia and New Zealand Neonatal Network; BAS, balloon atrial septostomy; CAPSNet, Canadian pediatric surgery network; CDH, congenital diaphragmatic hernia; CPQCC, California perinatal quality care collaborative; DHREAMS, diaphragmatic hernia research & exploration; Advancing Molecular Science; EA, esophageal atresia; FIP, focal intestinal perforation; HLHS, hypoplastic left heart syndrome; NEC, necrotizing enterocolitis; NICU, neonatal intensive care unit; NSW, New South Wales; OSHPD, office of statewide health planning and development; TAPVC, total anomalous pulmonary venous connection; TEF, tracheo-esophageal fistula; TGA, transposition of great arteries; VLBW, very low birth weight; VON, Vermont oxford network; VSD, ventricular septal defect.

### CDH

A total of 24 studies [10, 20–42] involving infants with CDH were included. Twelve studies were multi-centric [10,20,23,25,26,29,33,35,36,39,40,42] and 12 were single-center studies [21,22,24,27,28,30–32,34,37,38,41]. Four studies excluded infants with associated cardiac and/or chromosomal anomalies  [10,20,21,23].

Majority of studies reported in-hospital mortality as primary outcome while three studies reported mortality at 60 days and during infancy [20,26,42]. Most studies accounted for pre- and post-operative mortality in the analysis while one study [23] reported mortality only in infants undergoing surgical repair in either group. Kim et al. primarily evaluated thoracoscopic repair of CDH and reported 100% survival in both groups [32].

**Fig 2:**
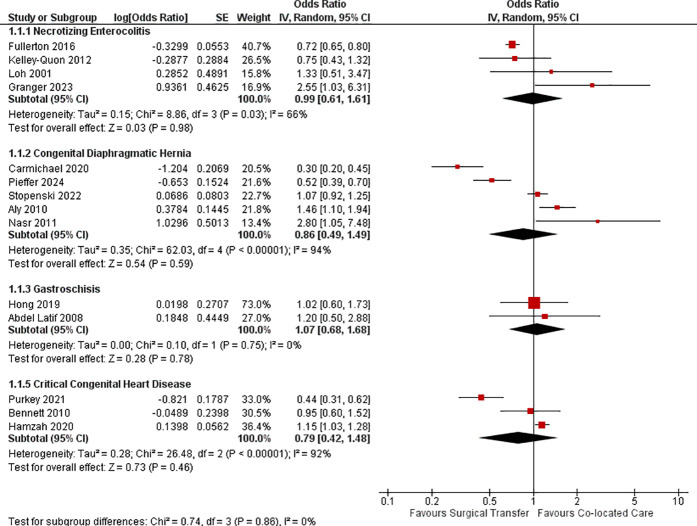
Forest plot for risk-adjusted mortality in surgical neonates.

**Fig 3:**
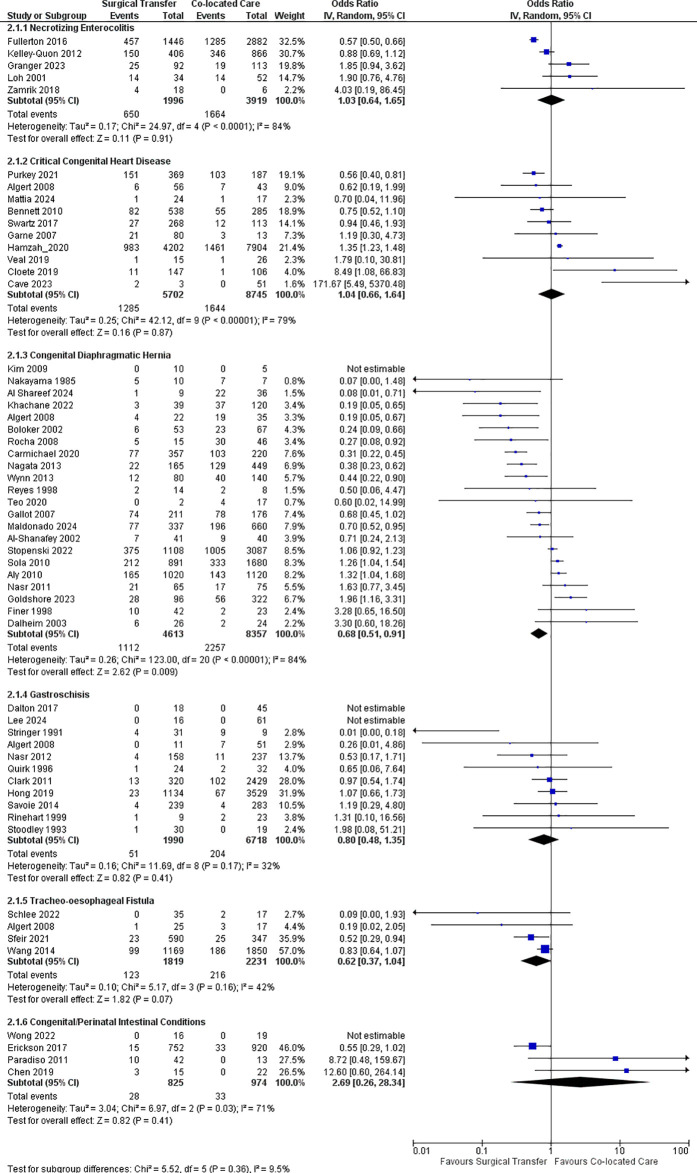
Forest plot for crude mortality in surgical neonates.

Risk-adjusted mortality was not significantly different between the transfer vs the co-located group [aOR 0.86 (0.49, 1.49), P = 0.59, I^2^ = 94%, 5 studies, 8366 infants [23,26,35,36,40] (Fig 2). Crude mortality was significantly lower in transfer vs co-located group infants [OR 0.68 (0.51, 0.91), P < 0.001, I^2^ = 84%, 22 studies, 12970 infants [10,20–24,26–35,37–42] (Fig 3). One study reported no significant difference in long-term neurodevelopmental outcome between the groups [41].

### CCHD

Eleven studies evaluated CCHD requiring cardiac surgery or interventional procedures during the neonatal period or primary hospitalization [20,43–52]. Six studies evaluated infants with TGA [20,44,46,48,51,52], two evaluated HLHS [47,49], three reported outcomes of more than one CCHD [43,45,50]. Eight studies were multicentric [20,43,45–47,49,50,52] and three were single center studies [44,48,51]. Five studies excluded infants with chromosomal or extra-cardiac anomalies [20,44–47].

In-hospital mortality was the primary outcome in three studies [46,47,52]. Four studies reported mortality at one year [20,46,49,51]. Other studies reported mortality at 90 [43,44] or 28 days [45,50]. Mortality before transfer was reported in only two studies [44,45]. The risk-adjusted and crude mortality at latest follow up was comparable between the transfer group and co-located group [aOR 0.79 (0.42, 1.48), P = 0.46, I^2^ = 92%, 3 studies,13485 infants [43,47,49]; OR 1.04 (0.66, 1.64), P = 0.87, I^2^ = 79%, 10 studies, 14447 infants [20,43–50,52].

### Acquired intestinal surgical conditions

Infants with proven NEC or FIP referred for exploratory laparotomy or primary peritoneal drainage were evaluated in five studies [7,53–56]. Four studies were multi-centric. Four studies exclusively reported on infants with surgical NEC while one [7] included infants with either NEC or FIP.

Two studies reported mortality only among infants who underwent surgical intervention [53,56] while other three reported mortality among all co-located and transfer group infants. In-hospital mortality was the primary outcome in four studies, while one study reported mortality at one year [54]. The risk-adjusted and crude mortality were comparable between the two groups [aOR 0.99 (0.61, 1.61), P = 0.98, I^2^ = 66%, 4 studies, 5891 infants [7,53–55]; OR 1.03 (0.64, 1.65), P = 0.91, I^2^ = 84%, 5 studies, 5915 infants [7,53–56]. In one of the included studies, [53] 19% of the surgical transfers happened before diagnoses of NEC. Excluding it from the analysis did not alter the overall effect. Two studies that reported neurodevelopmental outcomes at two years found no difference between the two groups [7,56] (S4 table).

### Gastroschisis

A total of 14 studies [20,57–69] were included of which seven were multi-centric [20,57,58,60,63,66,69] and seven were single center studies [59,61,62,64,65,67,68]. Two studies excluded infants with other anterior abdominal wall defects [65,66] and one excluded infants with complex gastroschisis [59]. One study included only antenatally diagnosed gastroschisis [63]. Risk-adjusted and crude mortality were not significantly different between the transfer and co-located group infants [aOR 1.07 (0.68,1.68), P = 0.78, I^2^ = 0, 2 studies, 5294 infants [57,60]; OR 0.80(0.48,1.35), P = 0.41, I^2^ = 32%, 11 studies, 8708 infants [20,58–60,62–68].

### EA/TEF

Four studies were included [20,70–72]; three were multi-centric [20,71,72]. One study excluded long gap EA [70]. Three studies reported mortality among all infants in co-located and transfer group [20,71,72] while one study reported outcomes only among infants that were operated [70].

Mortality was reported during primary hospitalization [72] and at three months [71] and one year [20,70]. Mortality at latest follow-up was comparable between transfer and co-located group [aOR 0.97 (0.39, 2.39), P = 0.95, one study, 937 infants [71]; OR 0.62 (0.37, 1.04), P = 0.07, I^2^ = 42%, 4 studies, 4050 infants [20,70–72].

### Congenital/perinatal intestinal conditions

Four studies were included [73–76]. Two studies were multi-centric [73,74] and two were single center studies [75,76]. Three studies evaluated infants with meconium ileus or perinatal intestinal perforation [73,75,76]; one study evaluated infants with intestinal atresia [74]. The primary outcome was in-hospital mortality in all these studies. Three studies reported mortality among all study infants, while one included only infants that underwent surgical repair [74]. In one study evaluating meconium ileus [75], nearly 50% of the infants initially designated for surgical repair were managed conservatively. In-hospital mortality was not significantly different between the study groups [OR 2.69 (0.26, 28.34), P = 0.41, I^2^ = 71%, 4 studies, 1799 infants [73–76].

### Other surgical conditions

Kancherla 2021 [77] and Algert 2008 [20] studied neural tube defects and Yang 2015 [78] reported outcomes of neonatal gastric perforation (S4 table).

### Assessment of study quality and certainty of evidence

Majority (n = 45/61) of the studies were judged as good quality based on assessment of the three domains a) selection of study population b) comparability of study cohorts c) outcome assessment and follow-up. 16 studies were rated low quality as they did not report mortality estimates adjusted for confounders and had unbalanced group sizes (Table 2).

**Table 2:** Risk of bias assessment.

Assessment using Newcastle- Ottawa Scale for cohort studies				
Name of the study	Selection of study cohorts	Comparabilityk,z of cohorts	Outcome assessments	Overall quality
**Congenital diaphragmatic hernia**				
Algert 2008 [20]	***	*	***	Good
Al- Shanafey 2002 [21]	***	*	***	Good
Al-Shareef 2022 [22]	***		***	Poor
Aly 2010 [23]	***	**	***	Good
Boloker 2002 [24]	***	*	***	Good
Bryner 2009 [25]	***	*	***	Good
Carmichael 2020 [26]	***	**	***	Good
Dalhiem 2003 [27]	***	*	***	Good
Finer 1998 [28]	***	*	***	Good
Gallot 2007 [29]	***	*	***	Good
Goldshore 2023 [30]	***		***	Poor
Khachane 2022 [31]	***	*	***	Good
Kim 2009 [32]	***	*	***	Good
Maldonado 2024 [10]	***	*	***	Good
Nagata 2013 [33] #	***		***	Poor
Nakayama 1985 [34]	***	*	***	Good
Nasr 2011 [35]	***	**	***	Good
Peiffer 2024 [36]	***	**	***	Good
Reyes 1998 [37]	***	*	***	Good
Rocha 2008 [38] #	***		***	Poor
Sola 2010 [39] #	***		***	Poor
Stopenski 2022 [40]	***	**	***	Good
Teo 2020 [41]	***		***	Poor
Wynn 2013 [42]	***	*	***	Good
**Congenital heart disease**				
Bennett 2010 [43]	***	**	***	Good
Cave 2023 [44]	***		***	Poor
Cloete 2019 [45]	***	*	***	Good
Garne 2007 [46] #	***		***	Poor
Hamzah 2020 [47]	***	**	***	Good
Mattia 2024 [48]	***	*	***	Good
Purkey 2021 [49]	***	**	***	Good
Swartz 2017 [50]	***	*	***	Good
Thomas 2023 [51]	***	*	***	Good
Veal 2019 [52]	***		***	Poor
**Necrotising enterocolitis and spontaneous intestinal perforation**				
Fullerton 2016 [53]	***	**	***	Good
Granger 2023 [7]	***	**	***	Good
Kelley-Quon 2012 [54]	***	**	***	Good
Loh 2001 [55]	***	**	***	Good
Zamrik 2018 [56]#	***		***	Poor
**Gastroschisis**				
Abdel Latif 2008 [57]	***	**	***	Good
Clark 2011 [58]	***		***	Poor
Dalton 2017 [59] #	***		***	Poor
Hong 2019 [60]	***	**	***	Good
Kandasamy 2010 [61]	***	*	***	Good
Lee 2024 [62] #	***		***	Poor
Nasr 2012 [63]	***	*	***	Good
Quirk 1996 [64]	***	*	***	Good
Rinehart 1999 [65] #	***		***	Poor
Savoie 2014 [66]	***	**	***	Good
Stoodley 1993 [67]	***	*	***	Good
Stringer 1991 [68] #	***		***	Poor
Youssef 2016 [69]	***	**	***	Good
**Tracheo-oesophageal fistula**				
Schlee 2022 [70]	***	*	***	Good
Sfeir 2021 [71]	***	**	***	Good
Wang 2014 [72]	***	*	***	Good
**Intestinal atresia/meconium peritonitis**				
Chen 2019 [73]	***	*	***	Good
Erickson 2017 [74]	***	*	***	Good
Paradiso 2011 [75] #	***		***	Poor
Wong 2023 [76]	***	*	***	Good
**Meningomyelocele**				
Kancherla 2021 [77]	***	**	***	Good
**Gastric perforation**				
Yang 2015 [78]	***	*	***	Good

Each of the above criteria was given one star if the following criteria were fulfilled.

Good quality: 3 or 4 stars in selection domain AND 1 or 2 stars in comparability domain AND 2 or 3 stars in outcome domain.

Fair quality: 2 stars in selection domain AND 1 or 2 stars in comparability domain AND 2 or 3 stars in outcome domain.

Poor quality: 0 or 1 star in selection domain OR 0 stars in comparability domain OR 0 or 1 star in outcome domain.

**Selection of study cohort:**

*** All studies fulfilled at least 3 out of the 4 following criteria:

a) Representativeness of the exposed cohort: The study cohorts were representative of surgical neonates in general population. Case mix included term and preterm infants, antenatally and postnatally diagnosed conditions, infants with and without congenital or chromosomal abnormalities and infants with a range of co-morbidities.

b) Selection of the non-exposed cohort: Transferred and inborn group of infants were selected from hospitals with different levels of perinatal and neonatal care.

c) Ascertainment of exposure: Information on birth location and centre of disposition ascertained from population level or hospital level databases in high-income countries.

d) Demonstration that outcome of interest was not present at start of study

**Comparability of cohorts:**

** study reported outcome estimates after adjusting for important confounders

* Group sizes were equal, but only crude mortality rates were reported

# Group sizes were unequal and only crude mortality rates were reported

**Outcome asssessments:**

*** All studies fulfilled the following three criteria:

a) Primary outcome of mortality was objective and abstracted through record linkage.

b) Adequate follow up period for mortality.

c) Completeness of follow up.

**Fig 4:**
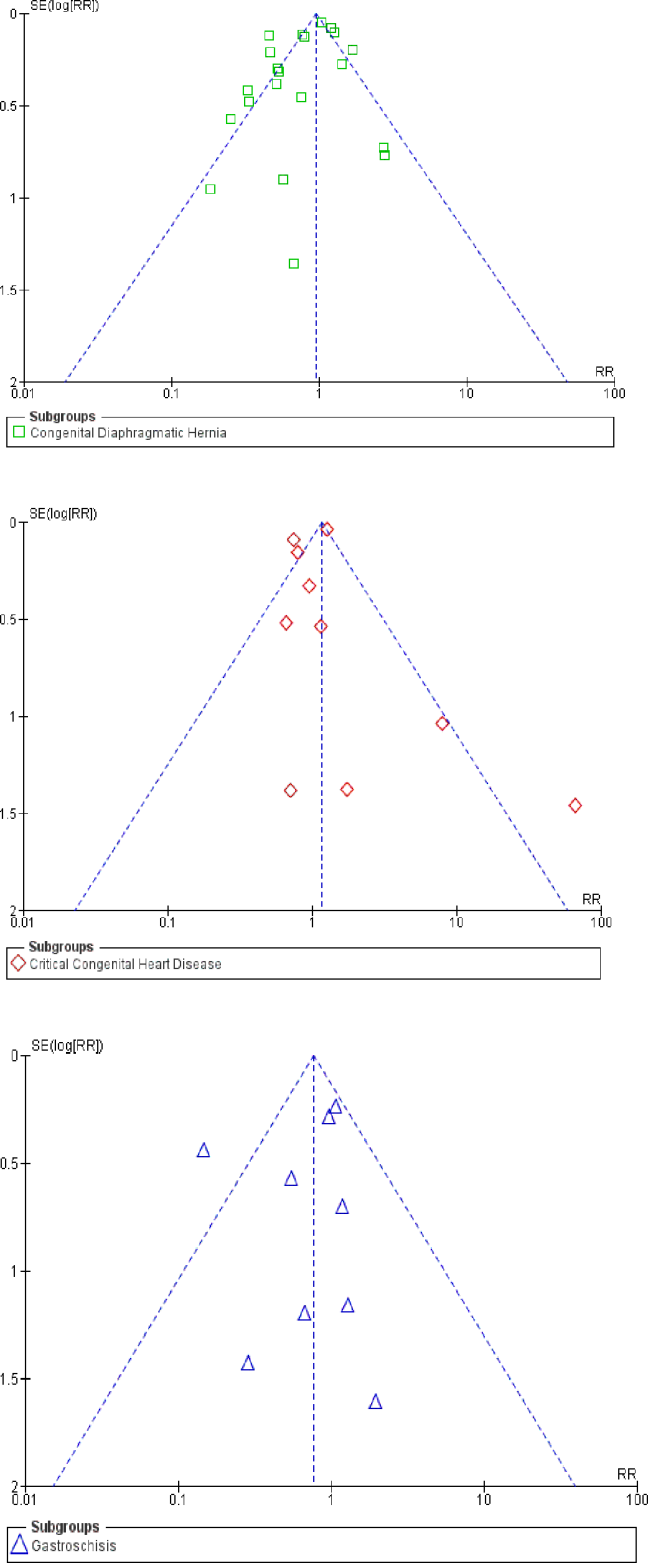
Funnel plot for assessment of publication bias.

The certainty of evidence was rated very low due to high risk of bias in observational studies, imprecision (wide 95% confidence intervals that included both benefit and harm) and inconsistency as evidenced by high statistical heterogeneity (Table 3). Publication bias was less likely given the visual impression of funnel plot symmetry (Fig 4).

**Table 3:** Certainty of Evidence.

Certainty assessment							Effect			
No of studies	Study design	Risk of bias	Inconsistency	Indirectness	Imprecision	Other considerations	Relative (95% CI)	Absolute (95% CI)	Certainty	Importance
**Risk adjusted mortality – Necrotizing Enterocolitis**										
4	Non-randomised studies	Serious	Serious	Not serious	Serious	None	**OR 0.99** (0.61 to 1.61)	**1 fewer per 1,000** (from 2 fewer to 1 fewer)	⨁○○○ Very low^a^	CRITICAL
**Risk adjusted mortality – Congenital Diaphragmatic Hernia**										
5	Non-randomised studies	Serious	Very serious	Not serious	Serious	None	**OR 0.86** (0.49 to 1.49)	**1 fewer per 1,000** (from 1 fewer to 0 fewer)	⨁○○○ Very low^a^	CRITICAL
**Risk adjusted mortality – Gastroschisis**										
2	Non-randomised studies	Serious	Not serious	Not serious	Serious	None	**OR 1.07** (0.68 to 1.68)	**1 fewer per 1,000** (from 2 fewer to 1 fewer)	⨁⨁○○ low^b^	CRITICAL
**Risk adjusted mortality – Critical Congenital Heart Disease**										
3	Non-randomised studies	Serious	Very serious	Not serious	Serious	None	**OR 0.79** (0.42 to 1.48)	**1 fewer per 1,000** (from 1 fewer to 0 fewer)	⨁○○○ Very low^a^	CRITICAL

CI, confidence interval; OR, odds ratio.

^a^Downgraded three levels due to imprecision (wide 95% CI that includes both benefit and harm), inconsistency (as evidenced by high statistical heterogeneity) and high risk of bias.

^b^Downgraded two levels due to imprecision (wide 95% CI that includes both benefit and harm) and high risk of bias.

### Sensitivity analyses

It is possible that temporal trends in transport related outcomes, severity of surgical condition, inclusion of low-quality studies and duplicate patient cohorts may impact the overall outcome estimates in our meta-analysis. Transport for neonatal surgery may putatively impact early postnatal outcomes than the outcomes through infancy.

We conducted five post-hoc sensitivity analyses: (1) excluding studies published before year 2000 (four studies each in CDH and gastroschisis). (2) excluding studies evaluating TGA among the CCHD category. (3) excluding studies of low quality. (4) including studies that only reported mortality at discharge. (5) to account for the possibility of double counting of infants across databases (Table 4). Our findings remained unchanged and robust to the sensitivity analyses.

**Table 4:** Sensitivity analysis.

Surgical condition	Outcome	No. of studies	Sample size (surgical transfer+ co-located care)	Odds ratio (95% CI)	I^2^ statistic
**Excluding studies published before January 2000**					
CDH	Mortality	19	12866(4547 + 8319)	0.67 [0.50, 0.90]	86%
Gastroschisis	Mortality	6	8454(1880 + 6574)	0.96 [0.68, 1.35]	0%
**Excluding studies evaluating TGA in the CCHD category**					
CCHD	Mortality	5	14119(8595 + 5524)	0.97 [0.58, 1.61]	88%
**Excluding low quality studies**					
CDH	Mortality	16	9242 (3435 + 5807)	0.66 [0.47, 0.93]	83%
CCHD	Mortality	7	14259 (5604 + 8655)	0.92 [0.58, 1.46]	82%
Gastroschisis	Mortality	6	5747 (1596 + 4151)	0.96 [0.64, 1.44]	0%
Congenital/perinatal intestinal conditions	Mortality	3	1744 (783 + 961)	1.82 [0.09, 36.06]	74%
Necrotising enterocolitis	Mortality	4	5891 (1978 + 3913)	1.00 [0.62, 1.60]	87%
**Including studies that reported mortality at hospital discharge**					
CDH	Mortality	20	12336 (4234 + 8102)	0.78 [0.60, 1.02]	77%
CCHD	Mortality	6	13645 (5194 + 8451)	1.12 [0.73, 1.71]	61%
Gastroschisis	Mortality	8	8646 (1979 + 6667)	0.83 [0.48, 1.42]	36%
Necrotising enterocolitis	Mortality	4	4643 (1590 + 3053)	1.29 [0.52, 3.23]	84%
**Excluding studies with population overlap**					
CDH^a^	Mortality	20	10342(3700 + 6642)	0.67 [0.48, 0.92]	83%
CCHD^b^	Mortality	8	13850(8541 + 5309)	1.19 [0.74, 1.90]	68%
Gastroschisis^c^	Mortality	8	8046(6329 + 1717)	0.74 [0.41, 1.35]	39%

CDH, congenital diaphragmatic hernia; CCHD, critical congenital heart disease; TGA, transposition of great arteries.

^a^The overlap between Aly 2010 and Sola 2010 (KIDS database) and that of Algert 2008 and Khachane 2022 (New South Wales database) could not be ruled out. Hence, Sola 2010 and Algert 2008 were excluded from this analysis

^b^The overlap between Purkey 2021 and Hamzah 2020 – (both evaluating patients with hypoplastic left heart syndrome from US database) could not be excluded. Hence Purkey 2021 was excluded from this analysis.

^c^The overlap of patients in Savoie 2014 and Dalton 2017 with that of Hong et al evaluating infants from the Vermont Oxford Network of 159 US neonatal units could not be ruled out. Hence, Savoie 2014 and Dalton 2017 were excluded from this analysis.

## Discussion

With a sample size of over 51,000 infants from total 20 HICs and spanning across a period of five decades, this is perhaps the first comprehensive and robust systematic review and meta-analysis evaluating the association between transport and death and disability in neonates with a range of major surgical conditions. Our assessment was restricted to HICs to ensure a comparable standard of care including transport for surgery for this high-risk cohort. Nearly 60% (36/61 studies) of the included studies extracted data from population-level databases or large collaborative neonatal or surgical networks, enhancing the validity of our findings. Our review captured survival beyond hospital discharge and through infancy, making the findings more relevant for infants with cardiac conditions requiring staged surgical repairs. Importantly, < 1% of infant deaths included in our analysis were due to palliation.

A recent systematic review of 19 cohort studies evaluated the association of birth-location with short-term outcomes for infants with gastroschisis, CDH and TEF [6]. Using a vote counting approach, they reported favourable outcomes with co-located care in seven studies and a lack of association of birth-place on outcomes in 12 studies. They misinterpreted the effect direction in three studies on CDH [20,26,33] and reported that birth in maternity unit with a co-located surgical center was associated with reduced mortality for CDH.

Reports from HICs indicate in-hospital mortality rates varying from 4–8% for gastroschisis [11], 12% for EA [79] and 25–30% for CCHD and surgical NEC [80,81]. Mortality ~70% has been reported in preterm very low birth weight infants with CDH [82]. Clinical risk prediction models may largely explain these disparities in survival and help standardize risk factors for mortality in various surgical conditions [83–87]. Many prediction models validated in contemporary surgical cohorts have not identified birth location as a significant risk factor for mortality [88–92]. Furthermore, mortality in CDH and surgical NEC has not reduced below 30% despite dedicated efforts [1,93,94]. These findings suggest that localisation of care may not independently influence mortality in most surgical infants.

A multi-center cohort study from the USA [2] evaluated survival in 790 infants with prenatally diagnosed HLHS after stage 1 palliation. The hospital length of stay, 30-day survival, or survival to discharge did not differ based on the distance from birth location to surgical center. In a Western Australian transport audit of 80 neonates with TGA transferred over ~3000 km distance to Melbourne for arterial switch repair, there was no mortality during transport and only one early post-operative death [8]. These two studies were not included in our review because they assessed the effect of transport distance rather than birth location, on mortality [2] or lacked a control group [8]. Nevertheless, their results provide further reassurance that transportation is unlikely to worsen outcomes in infants with critical surgical conditions.

Our findings that crude mortality of neonates with CDH was lower in the transfer group, warrants an explanation. Majority of these infants were likely diagnosed postnatally; such infants are known to have lesser pulmonary hypoplasia probably due to late onset of herniation during gestation [95].

Advocacy for a wider adoption of co-located care approach is often met with challenges. Parental choices, need for staying away from home during the vulnerable antenatal period, travel and cost constraints, surgeons’ preferences and insurance coverage patterns are known to influence regionalization practices for surgical neonates [96–98]. Furthermore, the discordance in availability of maternal and neonatal care and a surge in satellite NICUs around surgical centres may drive referral practices [99]. In the US, local care protocols for surgical neonates have been reported to vary significantly across states and lack consistency with surgical subspecialty standards endorsed by the American Academy of Pediatrics [100]. Thus, surgical transfers will continue to remain an important issue for healthcare providers and policy makers.

The limitations of our study need to be acknowledged. These include the potential for confounding by indication. Risk-adjusted mortality was reported in only 26% of the studies. The high statistical heterogeneity in our meta-analysis warrants caution in interpreting our findings. It may relate to the case mix from a wide range of patient populations studied. It is reassuring to note that our findings remained unchanged after excluding studies published before the year 2000, a potential source of heterogeneity. Inadvertent overlap of patients is a common occurrence in evidence synthesis involving public health records [101] and cannot be ruled out. Limited information was available on the impact of surgical transport on long-term neurodevelopment.

In summary, our systematic review and meta-analysis shows that transfer from the birth hospital to a facility for surgical intervention was not associated with increased risk of death in neonates with critical surgical conditions. Limited data was available on long-term disability. Considering the difficulties in conducting RCTs in this field, the findings from our comprehensive and robust systematic review provide the best available evidence for informing clinicians, parents, and health policy makers.

## Supporting information

S1 Checklist PRISMA 2020 checklist. (DOCX)

S1 File Search strategy. (DOCX)

S1 Table Additional study characteristics of included studies. (DOCX)

S2 Table Characteristics of excluded studies. (DOCX)

S3 Table Definition of co-located care and surgical transfer. (DOCX)

S4 Table Outcomes described in the systematic review. (DOCX)

## Abbreviations

CCHD: critical congenital heart disease
CDH: congenital diaphragmatic hernia
EA: esophageal atresia
HIC: high-income countries
HLHS: hypoplastic left heart syndrome
NEC: necrotizing enterocolitis
NICU: neonatal intensive care unit
OR: odds ratio
FIP: focal intestinal perforation
RCT: randomized controlled trial
TEF: tracheo-esophageal fistula
TGA: transposition of great arteries.
