# Supplementary material for: Association between transfer for surgery and mortality and disability among neonates in high income countries—A systematic review with meta-analysis
Source: PLoS One. 2025 Jul 31;20(7):e0327971. doi: 10.1371/journal.pone.0327971 (PMC12312895; doi:10.1371/journal.pone.0327971)
Supplement: S1 File — (DOCX) [file pone.0327971.s002.docx]

**S1. File.** **Search strategy**

**PubMed**

#1

((((((((((((((((((((((((((((((((((((((((((((((((((((((((((((((((((((((((((((((((((((((((diaphragmatic hernia[MeSH Terms]) OR (atresia, esophageal[MeSH Terms])) OR (fistula, tracheoesophageal[MeSH Terms])) OR (enterocolitis, necrotizing[MeSH Terms])) OR (necrotizing enterocolitis[MeSH Terms])) OR (intestinal perforation[MeSH Terms])) OR (gastroschisis[MeSH Terms])) OR (atresia, jejunal[MeSH Terms])) OR (atresia, intestinal[MeSH Terms])) OR (intestinal volvulus[MeSH Terms])) OR (volvulus[MeSH Terms])) OR (hirschsprung disease[MeSH Terms])) OR (great vessels transposition[MeSH Terms])) OR (transposition of great arteries[MeSH Terms])) OR (transposition of great vessels[MeSH Terms])) OR (hypoplastic left heart syndrome[MeSH Terms])) OR (atresia, pulmonary[MeSH Terms])) OR (atresia, pulmonary valve[MeSH Terms])) OR (coarctation, aortic[MeSH Terms])) OR (pulmonary stenosis[MeSH Terms])) OR (atresia, tricuspid[MeSH Terms])) OR (atresia, tricuspid valve[MeSH Terms])) OR (aortic stenosis[MeSH Terms])) OR (total anomalous pulmonary venous return[MeSH Terms])) OR (tapvr[MeSH Terms])) OR (truncus arteriosus[MeSH Terms])) OR (ebstein anomaly[MeSH Terms])) OR (congenital heart defects[MeSH Terms])) OR (defect, congenital heart[MeSH Terms])) OR (meningomyelocele[MeSH Terms])) OR (congenital hydrocephalus[MeSH Terms])) OR (hydrocephalus[MeSH Terms])) OR (defects, neural tube[MeSH Terms])) OR (encephalocele[MeSH Terms])) OR (congenital cystic adenomatoid malformation of lung[MeSH Terms])) OR (congenital cystic adenomatoid malformation[MeSH Terms])) OR (bronchopulmonary sequestration[MeSH Terms])) OR (bronchogenic cysts[MeSH Terms])) OR (reflux, vesicoureteral[MeSH Terms])) OR (vesicoureteral reflux[MeSH Terms])) OR (exstrophies, urinary bladder[MeSH Terms])) OR (bladder exstrophy[MeSH Terms])) OR (ureterocele[MeSH Terms])) OR (anal atresia[MeSH Terms])) OR (anus, imperforate[MeSH Terms])) OR (esophageal atresia[Title/Abstract])) OR (tracheoesophageal fistula[Title/Abstract])) OR (congenital diaphragmatic hernia[Title/Abstract])) OR (necrotizing enterocolitis[Title/Abstract])) OR (spontaneous intestinal perforation[Title/Abstract])) OR (gastroschisis[Title/Abstract])) OR (duodenal atresia[Title/Abstract])) OR (jejunal atresia[Title/Abstract])) OR (ileal atresia[Title/Abstract])) OR (meconium ileus[Title/Abstract])) OR (meconium peritonitis[Title/Abstract])) OR (intestinal malrotation[Title/Abstract])) OR (volvulus[Title/Abstract])) OR (Hirschsprung disease[Title/Abstract])) OR (Transposition of great vessels[Title/Abstract])) OR (Hypoplastic left heart syndrome[Title/Abstract])) OR (Interrupted aortic arch[Title/Abstract])) OR (Pulmonary atresia[Title/Abstract])) OR (Pulmonary stenosis[Title/Abstract])) OR (aortic stenosis[Title/Abstract])) OR (coarctation of aorta[Title/Abstract])) OR (tricuspid atresia[Title/Abstract])) OR (total anomalous pulmonary venous return[Title/Abstract])) OR (congenital heart disease[Title/Abstract])) OR (Ebstein anomaly[Title/Abstract])) OR (meningomyelocele[Title/Abstract])) OR (neural tube defects[Title/Abstract])) OR (encephalocele[Title/Abstract])) OR (hydrocephalus[Title/Abstract])) OR (congenital hydrocephalus[Title/Abstract])) OR (lung sequestration[Title/Abstract])) OR (cystic adenomatoid malformation[Title/Abstract])) OR (congenital pulmonary alveolar malformation[Title/Abstract])) OR (congenital lobar emphysema[Title/Abstract])) OR (posterior urethral valve[Title/Abstract])) OR (bronchogenic cysts[Title/Abstract])) OR (pelviureteric junction obstruction[Title/Abstract])) OR (ureteropelvic junction obstruction[Title/Abstract])) OR (vesicoureteral reflux[Title/Abstract])) OR (exstrophy bladder[Title/Abstract])) OR (ureterocele[Title/Abstract])) OR (anal atresia[Title/Abstract])) OR (anorectal malformations[Title/Abstract])) OR (imperforate anus[Title/Abstract])

#2

(((((((((((((((((((((((birth center[MeSH Terms]) OR (birth place[MeSH Terms])) OR (birth hospital[Title/Abstract])) OR (inborn[Title/Abstract])) OR (outborn[Title/Abstract])) OR (transferred in[Title/Abstract])) OR (site of delivery[Title/Abstract])) OR (specialty center[Title/Abstract])) OR (site of delivery[Title/Abstract])) OR (outside delivery[Title/Abstract])) OR (hospital transfer[Title/Abstract])) OR (specialist center[Title/Abstract])) OR (birth location[Title/Abstract])) OR (birth place[Title/Abstract])) OR (birth center[Title/Abstract])) OR (place of birth[Title/Abstract])) OR (outside hospital[Title/Abstract])) OR (co-located hospital[Title/Abstract])) OR (delivered outside[Title/Abstract])) OR (tertiary centers[Title/Abstract])) OR (hospital of birth[Title/Abstract])) OR (in-house[Title/Abstract])) OR (on-site[Title/Abstract])) OR (intrahospital transfer[Title/Abstract])

#3

((((infant, newborn[MeSH Terms]) OR (neonate[MeSH Terms])) OR (neonat*[Title/Abstract])) OR (newborn*[Title/Abstract])) OR (infant*[Title/Abstract])

#1 AND #2 AND #3 == 798

**Embase <1980 to January 24, 2025>**

1 Congenital diaphragmatic hernia.mp. or congenital diaphragm hernia/ 9448

2 esophageal atresia.mp. or esophagus atresia/ 7083

3 tracheoesophageal fistula.mp. or tracheoesophageal fistula/ 7916

4 necrotizing enterocolitis/ 17172

5 intestine perforation/ or spontaneous intestinal perforation.mp. 17272

6 gastroschisis.mp. or gastroschisis/ 4924

7 duodenal atresia.mp. or duodenum atresia/ 1726

8 intestine atresia/ or jejunal atresia.mp. 2811

9 ileal atresia.mp. 410

10 intestinal malrotation.mp. or intestine malrotation/ 2344

11 volvulus.mp. or intestine volvulus/ 13518

12 meconium ileus.mp. or meconium ileus/ 1965

13 meconium peritonitis.mp. or meconium peritonitis/ 756

14 Hirschsprung disease.mp. or Hirschsprung disease/ 8896

15 Transposition of great vessels.mp. or great vessels transposition/ 10338

16 Hypoplastic left heart syndrome.mp. or hypoplastic left heart syndrome/ 8993

17 Interrupted aortic arch.mp. or aortic arch interruption/ 1827

18 Pulmonary atresia.mp. or pulmonary valve atresia/ 7662

19 aorta coarctation/ or Coarctation of aorta.mp. 12898

20 Pulmonary stenosis.mp. or pulmonary valve stenosis/ 11936

21 tricuspid atresia.mp. or tricuspid valve atresia/ 3393

22 aortic atresia.mp. 565

23 aortic stenosis.mp. or aortic stenosis/ 45577

24 total anomalous pulmonary venous connection.mp. or lung vein drainage anomaly/ 5001

25 total anomalous pulmonary venous return.mp. 638

26 truncus arteriosus.mp. or arterial trunk/ 3576

27 congenital heart disease.mp. or congenital heart disease/ 77457

28 duct dependent cardiac lesions.mp. 5

29 Ebstein anomaly.mp. or Ebstein anomaly/ 4374

30 meningomyelocele.mp. or meningomyelocele/ 8801

31 hydrocephalus/ or congenital hydrocephalus/ or hydrocephalus.mp. 59212

32 neural tube defects.mp. or neural tube defect/ 17122

33 encephalocele.mp. or encephalocele/ 5421

34 lung sequestration/ or cystic adenomatoid malformation/ or Congenital pulmonary alveolar malformation.mp. 4749

35 cystic adenomatoid malformation.mp. 2149

36 lung sequestration.mp. or lung sequestration/ 3474

37 lung emphysema/ or congenital lobar emphysema.mp. 15122

38 bronchogenic cysts.mp. or lung cyst/ 5845

39 Posterior urethral valve.mp. 953

40 pelviureteric junction obstruction.mp. or ureteropelvic junction obstruction/ 5095

41 vesicoureteral reflux.mp. or vesicoureteral reflux/ 15181

42 exstrophy bladder.mp. or bladder exstrophy/ 3105

43 ureterocele.mp. or ureterocele/ 2048

44 anus atresia/ 4034

45 anorectal malformations.mp. or anorectal malformation/ 4339

46 1 or 2 or 3 or 4 or 5 or 6 or 7 or 8 or 9 or 10 or 11 or 12 or 13 or 14 or 15 or 16 or 17 or 18 or 19 or 20 or 21 or 22 or 23 or 24 or 25 or 26 or 27 or 28 or 29 or 30 or 31 or 32 or 33 or 34 or 35 or 36 or 37 or 38 or 39 or 40 or 41 or 42 or 43 or 44 or 45 373850

47 birth hospital.mp. 399

48 inborn.mp. 25641

49 outborn.mp. 776

50 specialty center.mp. 397

51 site of delivery.mp. 1711

52 outside delivery.mp. 6

53 transfer*.mp. 1171011

54 hospital transfer.mp. 1798

55 specialist center.mp. 382

56 place of birth.mp. 3262

57 birth place.mp. 418

58 birth location.mp. or birth setting/ 397

59 birth center.mp. 446

60 transferred in.mp. 4442

61 delivered outside.mp. 323

62 outside hospital.mp. 5142

63 co-located hospital.mp. 5

64 (tertiary centers or tertiary centre).mp. 13417

65 Hospital of birth.mp. 1332

66 in-house.mp. 48426

67 on-site.mp. 37235

68 intrahospital.mp. 2064

69 47 or 48 or 49 or 50 or 51 or 52 or 53 or 54 or 55 or 56 or 57 or 58 or 59 or 60 or 61 or 62 or 63 or 64 or 65 or 66 or 67 or 68 1301899

70 Infant*.mp. or infant/ 1011215

71 newborn.mp. or newborn/ 717709

72 neonate*.mp. 156043

73 70 or 71 or 72 1438424

74 46 and 69 and 73 1252

**CINAHL**

S1 MW Congenital diaphragmatic hernia 935

S2 MW esophageal atresia 675

S3 MW tracheoesophageal fistula 609

S4 MW necrotizing enterocolitis 2,517

S5 MW intestine perforation 116

S6 MW spontaneous intestinal perforation 42

S7 MW gastroschisis 506

S8 MW duodenal atresia 7

S9 MW intestine atresia 5

S10 MW jejunal atresia 0

S11 MW ileal atresia 0

S12 MW intestinal malrotation 0

S13 MW intestine volvulus 49

S14 MW meconium ileus 0

S15 MW meconium peritonitis 44

S16 MW Hirschsprung disease 825

S17 MW Transposition of great vessels 47

S18 MW Hypoplastic left heart syndrome 758

S19 MW Interrupted aortic arch 0

S20 MW Pulmonary atresia 268

S21 MW Coarctation of aorta 286

S22 MW Pulmonary stenosis 758

S23 MW tricuspid atresia 128

S24 MW aortic atresia 29

S25 MW aortic stenosis 6,718

S26 MW total anomalous pulmonary venous connection 0

S27 MW total anomalous pulmonary venous return 0

S28 MW truncus arteriosus 101

S29 MW congenital heart disease 773

S30 MW duct dependent cardiac lesions 0

S31 MW Ebstein anomaly 0

S32 MW meningomyelocele 0

S33 MW hydrocephalus 3,288

S34 MW congenital hydrocephalus 25

S35 MW neural tube defects 2,937

S36 MW encephalocele 0

S37 MW lung sequestration 0

S38 MW cystic adenomatoid malformation 226

S39 MW Congenital pulmonary alveolar malformation 0

S40 MW congenital lobar emphysema 0

S41 MW lung emphysema 1,117

S42 MW bronchogenic cysts 3

S43 MW Posterior urethral valve 0

S44 MW pelviureteric junction obstruction 0

S45 MW vesicoureteral reflux 1,021

S46 MW exstrophy bladder 254

S47 MW ureterocele 0

S48 MW anus atresia 17

S49 MW anorectal malformations 124

S50 S1 OR S2 OR S3 OR S4 OR S5 OR S6 OR S7 OR S8 OR S9 OR S10 OR S11 OR S12 OR S13 OR S14 OR S15 OR S16 OR S17 OR S18 OR S19 OR S20 OR S21 OR S22 OR S23 OR S24 OR S25 OR S26 OR S27 OR S28 OR S29 OR S30 OR S31 OR S32 OR S33 OR S34 OR S35 OR S36 OR S37 OR S38 OR S39 OR S40 OR S41 OR S42 OR S43 OR S44 OR S45 OR S46 OR S47 OR S48 OR S49 23,954

S51 MW birth hospital 504

S52 MW inborn 8,787

S53 MW outborn 0

S54 MW speciality center 0

S55 MW site of delivery 8

S56 MW outside delivery 0

S57 MW transfer 14,222

S58 MW hospital transfer 1,134

S59 MW speciality center 0

S60 MW place of birth 1,206

S61 MW birth location 0

S62 MW birth center 97

S63 MW transferred in 0

S64 MW delivered outside 0

S65 MW outside hospital 0

S66 MW co-located hospital 0

S67 MW tertiary center 9

S68 MW hospital of birth 504

S69 MW in-house 0

S70 MW on-site 2,261

S71 MW intrahospital 1,372

S72 S51 OR S52 OR S53 OR S54 OR S55 OR S56 OR S57 OR S58 OR S59 OR S60 OR S61 OR S62 OR S63 OR S64 OR S65 OR S66 OR S67 OR S68 OR S69 OR S70 OR S71 27,010

S73 MW infant 311,949

S74 MW newborn 151,697

S75 MW neonate 1

S76 S73 OR S74 OR S75 312,834

S77 S50 AND S72 AND S76 65

**# Web of Science Search Strategy (v0.1)**

# Searches:

1: Congenital diaphragmatic hernia (Keyword Plus ®) Results: 1439

2: esophageal atresia (Keyword Plus ®) Results: 874

3: tracheoesophageal fistula (Keyword Plus ®) Results: 1093

4: necrotizing enterocolitis (Keyword Plus ®) Results: 4311

5: intestine perforation (Keyword Plus ®) Results: 31

6: spontaneous intestinal perforation (Keyword Plus ®) Results: 72

7: gastroschisis (Keyword Plus ®) Results: 631

8: duodenal atresia (Keyword Plus ®) Results: 97

9: intestine atresia (Keyword Plus ®) Results: 6

10: jejunal atresia (Keyword Plus ®) Results: 34

11: ileal atresia (Keyword Plus ®) Results: 32

12: intestine malrotation (Keyword Plus ®) Results: 10

13: intestine volvulus (Keyword Plus ®) Results: 16

14: meconium ileus (Keyword Plus ®) Results: 147

15: meconium peritonitis (Keyword Plus ®) Results: 53

16: Hirschsprung disease (Keyword Plus ®) Results: 877

17: Transposition of great vessels (Keyword Plus ®) Results: 0

18: Transposition of great vessels (All Fields) Results: 792

19: hypoplastic left heart syndrome (Keyword Plus ®) Results: 34

20: Interrupted aortic arch (Keyword Plus ®) Results: 102

21: Pulmonary atresia (Keyword Plus ®) Results: 590

22: Coarctation of aorta (Keyword Plus ®) Results: 1

23: Pulmonary stenosis (Keyword Plus ®) Results:1087

24: tricuspid atresia (Keyword Plus ®) Results: 357

25: aortic atresia (Keyword Plus ®) Results: 219

26: aortic stenosis. (Keyword Plus ®) Results: 4430

27: total anomalous pulmonary venous connection (Keyword Plus ®) Results: 0

28: total anomalous pulmonary venous connection (All Fields) Results: 817

29: total anomalous pulmonary venous return (All Fields) Results: 430

30: truncus arteriosus (Keyword Plus ®) Results: 220

31: congenital heart disease (Keyword Plus ®) Results: 9524

32: duct dependent cardiac lesions (Keyword Plus ®) Results: 0

33: duct dependent cardiac lesions (All Fields) Results: 31

34: Ebstein anomaly (Keyword Plus ®) Results: 0

35: Ebstein anomaly (All Fields) Results: 1478

36: hydrocephalus (Keyword Plus ®) Results: 7410

37: congenital hydrocephalus (Keyword Plus ®) Results:361

38: neural tube defects (Keyword Plus ®) Results: 5275

39: encephalocele (Keyword Plus ®) Results: 336

40: lung sequestration (Keyword Plus ®) Results: 306

41: neural tube defect (Keyword Plus ®) Results: 5275

42: cystic adenomatoid malformation (Keyword Plus ®) Results: 683

43: congenital lobar emphysema (Keyword Plus ®) Results: 49

44: bronchogenic cysts (Keyword Plus ®) Results: 245

45: Posterior urethral valve (Keyword Plus ®) Results: 359

46: ureteropelvic junction obstruction (Keyword Plus ®) Results: 818

47: vesicoureteral reflux (Keyword Plus ®) Results: 2113

48: bladder exstrophy (Keyword Plus ®) Results: 300

49: ureterocele (Keyword Plus ®) Results: 81

50: anus atresia (Keyword Plus ®) Results: 43

51: anorectal malformation (Keyword Plus ®) Results:496

52: #51 OR #50 OR #49 OR #48 OR #43 OR #44 OR #45 OR #46 OR #47 OR #39 OR #40 OR

#41 OR #42 OR #34 OR #32 OR #33 OR #35 OR #36 OR #37 OR #38 OR #31 OR #30 OR #29

OR #28 OR #27 OR #26 OR #25 OR #24 OR #23 OR #22 OR #21 OR #20 OR #19 OR #18 OR

#17 OR #16 OR #15 OR #14 OR #13 OR #12 OR #11 OR #10 OR #9 OR #8 OR #7 OR #6 OR

#5 OR #4 OR #3 OR #2 OR #1 Results: 45548

53: birth hospital (Keyword Plus ®) Results: 747

54: inborn (Keyword Plus ®) Results: 2912

55: inborn (Keyword Plus ®) Results: 2912

56: outborn (Keyword Plus ®) Results: 12

57: speciality center (Keyword Plus ®) Results: 49

58: site of delivery (Keyword Plus ®) Results: 10

59: outside delivery (Keyword Plus ®) Results: 1

60: transfer (Keyword Plus ®) Results: 421462

61: hospital transfer (Keyword Plus ®) Results: 190

62: specialist center (Keyword Plus ®) Results: 25

63: place of birth (Keyword Plus ®) Results: 6

64:birthlocation(KeywordPlus®) Results: 24

65: birth lcenter (Keyword Plus ®) Results: 0

66: birth center (Keyword Plus ®) Results: 326

67: transferred in (Keyword Plus ®) Results: 19596

68: delivered outside (Keyword Plus ®) Results: 0

69: outside hospital (Keyword Plus ®) Results: 0

70: co located hospital (Keyword Plus ®) Results: 0

71: co located hospital (Topic) Results: 618

72: tertiary center (Keyword Plus Results: 888

73: hospital of birth (Keyword Plus ®) Results: 62

74: in-house (Keyword Plus ®) Results: 1535

75: on-site (Keyword Plus ®) Results: 1071

76: intrahospital (Keyword Plus ®) Results: 268

77: #53 OR #54 OR #55 OR #56 OR #57 OR #58 OR #65 OR #64 OR #63 OR #62 OR #61 OR

#60 OR #59 OR #71 OR #70 OR #69 OR #68 OR #67 OR #66 OR #76 OR #75 OR #74 OR #73

OR #72 Results: 429811

78: infant (Keyword Plus ®) Results: 143587

79: newborn (Keyword Plus ®) Results: 23204

80: neonate (Keyword Plus ®) Results: 1472

81: #78 OR #79 OR #80 Results: 159133

82: #52 AND #77 AND #81 Results: 26
