# Supplementary material for: Association between transfer for surgery and mortality and disability among neonates in high income countries—A systematic review with meta-analysis
Source: PLoS One. 2025 Jul 31;20(7):e0327971. doi: 10.1371/journal.pone.0327971 (PMC12312895; doi:10.1371/journal.pone.0327971)
Supplement: S1 Table — (DOCX) [file pone.0327971.s003.docx]

**S1 Table: Additional characteristics of included studies**

|  | Mortality before surgery | Mortality before transfer | Time of transfer | Survived without Surgery | Mortality related to palliation | Extracorporeal membrane oxygenation |
| --- | --- | --- | --- | --- | --- | --- |
| **Congenital Diaphragmatic Hernia** | | | | | | |
| Algert 2008^20^ | 16/35 Co-located infants vs  2/22 transfer infants | - | - | - | - | - |
| Al Shareef 2024^22^ | 19/23 total deaths |  |  |  |  | 1/45 total infants |
| Aly 2010^23^ | 33/171 Co-located infants vs 56/254 transfer infants on ECMO | - | < 8 days | 458/2140 total infants |  | 425/2140 total infants |
| Boloker 2002^24^ | 23/67 Co-located infants vs 6/53 transfer infants |  |  |  |  | 6/67 Co-located infants vs 10/53 transfer infants |
| Carmichael 2020^26^ | 39 /180 total deaths | 82 /180 total deaths |  |  |  |  |
| Dalheim 2003^27^ |  |  | 17/26 (transferred within first 24 hours) |  |  | 25/50 total infants |
| Finer 1998^28^ |  |  |  | 13/23 Co-located infants |  | 38/65 total infants |
| Goldshore 2023^30^ |  |  |  |  |  | 32% of 418 total infants |
| Khachane 2021^31^ | 31/159 total infants |  |  |  |  | 2/159 total infants |
| Kim 2009^32^ |  |  |  |  |  | 2/15 total infants |
| Maldonado 2024^10^ |  | 95/659 transfer infants | Median of 4 days |  |  | 54/660 Co-located infants vs  28/337 transfer infants |
| Nagata 2013^33^ |  |  |  | 84/614 total infants |  | 43/614 total infants |
| Nakayama 1985^34^ | 6/17 total infants |  | < 24 hours of age |  |  |  |
| Nasr 2011^35^ |  |  |  |  |  | 5/75 Co-located infants vs  10/65 transfer infants |
| Pieffer 2024^36^ |  |  |  |  |  | 136/1314 infants |
| Reyes 1998^37^ | 2/22 total infants |  |  |  |  | 4/22 total infants |
| Rocha 2008^38^ | 18/61 total infants |  |  |  |  |  |
| Sola 2010^39^ |  |  | <8 days |  |  | 344/2571 total infants |
| Stopenski 2021^40^ |  |  |  |  |  | 30.9% of Co-located infants  vs  31.5% of transfer infants |
| Teo 2020^41^ | 4/19 total infants |  |  |  |  |  |
| Wynn 2013^42^ |  |  | <1week |  |  | 70/220 total infants |
| **Critical Congenital Heart Disease** | | | | | | |
| Algert 2008^20^  (TGA) | 2/43 Co-located infants vs  2/56 transfer infants |  |  |  |  |  |
| Cave 2023^44^ |  | 2/3 transfer infants |  | 9/51 CC infants |  |  |
| Cloete 2018^45^ | 1/1 Co-located infant death | 10/11 transfer infant deaths |  |  |  |  |
| Garne 2007^46^ | 10/93 total infants |  |  |  | 8/93 total infants |  |
| Hamzah 2020^47^ |  |  | <28 days of age |  |  | 1523/18866 total infants |
| Purkey 2021^49^ | 107/556 total infants |  |  |  |  |  |
| Swartz 2017^50^ | 6/381 total infants (3 neonates palliated) |  | Within the first week  (Median of 4 days) |  |  | 1/113 Co-located infants vs 3/268 transfer infants |
| Thomas 2023^51^ |  |  | Median of 10.3 hours | 4/96 total infants did not require surgery in first admission |  |  |
| Veal 2019^52^ |  |  | Median of 92 minutes |  |  |  |
| **Esophageal Atresia/Tracheo-esophageal fistula** | | | | | | |
| Algert 2008^20^ | 2/17 Co-located infants |  |  |  |  |  |
| Schlee 2022^70^ | 1/57 total infants | - | - | - | -- | - |
| Sfeir 2021^71^ | 13/1008 total infants | - |  | - | - | - |
| Wang 2014^72^ | 14% of 1675 infants that did not undergo any surgical procedure | - | Repair within 24 hours of age in 89% of total infants | - | - | - |
| **Gastroschisis** | | | | | | |
| Algert 2008^20^ | 1/36 Co-located infants |  |  |  |  |  |
| Clark 2010^58^ | - | - | - | - | 22/115 total deaths | - |
| Hong 2018^60^ | 23/4663 (18/3529 Co-located infants vs 5/1134 transfer infants) | Nil |  | - | - | - |
| Kandasamy 2010^61^ |  |  |  |  | 1/3 total deaths |  |
| Lee 2024^62^ |  |  | Median of 5 hours |  |  |  |
| Savoie 2014^66^ | 1/8 total deaths | - | - | - | -- | - |
| **Intestinal atresia/Meconium peritonitis** | | | | | | |
| Chen 2019^73^ | - | - | - | 3/37 total infants managed conservatively | - | - |
| Paradiso 2011^75^ |  |  | Median of 6 days | 10/13 Co-located infants vs 22/42 transfer infants treated with gastrograffin enema |  |  |
| Wong 2023^76^ | - | - | - | 5/35 total infants | 1/2 total deaths | - |
| **Necrotizing Enterocolitis** | | | | | | |
| Granger 2022^7^ |  | None | Referral within 24 hrs of onset of symptoms in 80% of transferred cases | 41% of transfer infants and 54% of Co-located infants with NEC managed conservatively | 25% of transfer infants and 5% of Co-located infants with NEC were pan-enteric and were palliated | - |
| Loh 2001^55^ | 3/52 Co-located infants vs  11/72 transfer infants | 10/72 transfer infants | 23.3 vs 25.7 days (age at onset of NEC) | 14/52 Co-located infants did not require surgery and 28/72 infants – not transferred and did not undergo surgery | - | - |
| **Neural Tube Defects** | | | | | | |
| Algert 2008^20^ | 1/10 transfer infants |  |  |  |  |  |

ECMO- Extra corporeal membrane oxygenation, NEC- Necrotizing enterocolitis
