# Supplementary material for: Association between transfer for surgery and mortality and disability among neonates in high income countries—A systematic review with meta-analysis
Source: PLoS One. 2025 Jul 31;20(7):e0327971. doi: 10.1371/journal.pone.0327971 (PMC12312895; doi:10.1371/journal.pone.0327971)
Supplement: S2 Table — (DOCX) [file pone.0327971.s004.docx]

**S2 Table: Characteristics of excluded studies**

| **Study** | **Reason for exclusion** |
| --- | --- |
| **Congenital Diaphragmatic Hernia** | |
| Andrew 2001[1] | Mortality reported for antenatally vs postnatally diagnosed CDH, rather than inborn vs transferred status |
| Bojanic 2015[2] | Mortality reported for local versus remote transfers |
| Jancelewicz 2018[3] | Modelled hospital transfer as a variable to predict ECMO risk stratification in infants with CDH |
| Lazar 2011[4] | Mortality was compared between prenatally diagnosed vs postnatally diagnosed CDH infant groups |
| Pusic 1995[5] | Population overlap with Al-Shanafey 2002 |
| Putnam 2016[6] | Did not report mortality |
| Vasudev 2023[7] | Study conducted in middle-income country (India) |
| Wilson 1992[8] | Compared delayed versus immediate surgical repair for CDH |
| **Critical Congenital Heart Disease** | |
| Anderson 2013[9] | Mortality among transferred infants with TGA was not reported |
| Chee 2022[10] | Mortality predominantly from palliative care in HLHS. Did not report mortality by birth location |
| Gould 2003[11] | Compared PDA ligation in NICU at Childrens Hospital of Philadelphia versus other hospital NICUs |
| Karamlou 2010[12] | Compared mortality between rural and urban teaching hospital. Mortality also included patients that received comfort care |
| Kunde 2021[13] | Study conducted in middle-income setting (India) |
| Martini 2021[14] | Compared bedside PDA ligation in NICU vs ligation after transfer to a cardiac centre. |
| Morris 2014[15] | Mortality in HLHS was reported with respect to the time taken to transfer infants to cardiac surgical center |
| Patel 2022[16] | Compared mortality in infants with HLHS born <5 or > 5 miles away from surgical center |
| Purkey 2022[17] | Same cohort as Purkey 2021 |
| Wong 2013[18] | Compared PDA ligation in a non-cardiac centre versus cardiac centre. |
| Wu 2020[19] | Low income setting (China) |
| **Gastroschisis** | |
| Apfeld 2017[20] | Reported mortality by the level of NICU care |
| Du 2014[21] | Middle income setting (China) |
| Kitchanan 2000[22] | Population overlap with Kandasamy 2010 |
| Machaea 2023[23] | Middle income setting (South Africa) |
| Muniz 2021[24] | Middle income setting (Brazil) |
| Murphy 2007[25] | Mortality among antenatally diagnosed and postnatally diagnosed gastroschisis were reported. |
| Singh 2003[26] | Compared mortality between infants born near versus far from the tertiary center. |
| Vilela 2001[27] | Middle income setting (Brazil) |
| **Surgical Necrotising Enterocolitis** | |
| Murthy 2014[28] | Mortality among transferred versus non -transferred infants with surgical NEC was not reported. |
| **Congenital Intestinal Conditions** | |
| Gibson 2024[29] | 14% of patients with malrotation of volvulus of the small intestine were > 1 year of age |
| Jiang 2019[30] | Middle income setting (China) |
| Politis 2019[31] | 25% of infants with malrotation were > 1 year at age at surgery |
| **Tracheo-esophageal fistula /esophageal atresia** | |
| Zouari 2023[32] | Low income country (Tunisia) |
| **Others** | |
| Besendorfer 2024[33] | Compared outcomes of infants transferred to university hospital for surgery with those operated at hospitals affiliated to perinatal center. |
| Frassanito 2021[34] | Mortality among transferred infants with post hemorrhagic hydrocephalus was not reported |
| Kancherla 2023[35] | Same cohort as in Kancherla 2021. |
| Naicker 2023[36] | Middle income setting. |
| Nelson 2005[37] | 45% infants were discharged from birth hospital without bladder surgery |
| Parodi 2020[38] | Study on post haemorrhagic hydrocephalus. |
| Ugwu 2013[39] | Low-income setting (Nigeria). |

CDH: Congenital diaphragmatic hernia; ECMO: Extracorporeal membrane oxygenation; HLHS: Hypoplastic left heart syndrome; NICU: Neonatal intensive care unit; PDA: Patent ductus arteriosus; NEC: Necrotizing enterocolitis; TGA: Transposition of Great Arteries

1. Andrews R. Outcome of staged reconstructive surgery for hypoplastic left heart syndrome following antenatal diagnosis. Archives of Disease in Childhood. 2001 Dec 1;85(6):474–7.

2. Bojanić K, Pritišanac E, Luetić T, Vuković J, Sprung J, Weingarten TN, et al. Survival of outborns with congenital diaphragmatic hernia: the role of protective ventilation, early presentation and transport distance: a retrospective cohort study. BMC Pediatr. 2015 Dec;15(1):155.

3. Jancelewicz T, Brindle ME, Harting MT, Tolley EA, Langham MR, Lally PA, et al. Extracorporeal Membrane Oxygenation (ECMO) Risk Stratification in Newborns with Congenital Diaphragmatic Hernia (CDH). Journal of Pediatric Surgery. 2018 Oct;53(10):1890–5.

4. Lazar DA, Cass DL, Rodriguez MA, Hassan SF, Cassady CI, Johnson YR, et al. Impact of prenatal evaluation and protocol-based perinatal management on congenital diaphragmatic hernia outcomes. Journal of Pediatric Surgery. 2011 May;46(5):808–13.

5. Pusic AL, Giacomantonio M, Pippus K, Rees E, Gillis DA. Survival in neonatal congenital hernia without extracorporeal membrane oxygenation support. Journal of Pediatric Surgery. 1995 Aug;30(8):1188–90.

6. Putnam LR, Harting MT, Tsao K, Morini F, Yoder BA, Luco M, et al. Congenital Diaphragmatic Hernia Defect Size and Infant Morbidity at Discharge. Pediatrics. 2016 Nov;138(5):e20162043.

7. Vasudev RB, Kumar N, Gadgade BD, Radhakrishna V, Basavaraju M, Anand A. Factors Contributing to Mortality in Neonates with Congenital Diaphragmatic Hernia and Eventration. African Journal of Paediatric Surgery [Internet]. 2023 Jan 4 [cited 2024 Dec 10]; Available from: https://journals.lww.com/10.4103/ajps.ajps_165_21

8. Wilson JM, Lund DP, Lillehei CW, O’Rourke PP, Vacanti JP. Delayed repair and preoperative ECMO does not improve survival in high-risk congenital diaphragmatic hernia. Journal of Pediatric Surgery. 1992 Mar;27(3):368–75.

9. Anderson BR, Ciarleglio AJ, Hayes DA, Quaegebeur JM, Vincent JA, Bacha EA. Earlier Arterial Switch Operation Improves Outcomes and Reduces Costs for Neonates With Transposition of the Great Arteries. Journal of the American College of Cardiology. 2014 Feb;63(5):481–7.

10. Chee YH, Dunning-Davies B, Singh Y, Yates R, Kelsall W. Managing CHD in Tertiary NICU in Collaboration with a Cardiothoracic Center. Pediatr Cardiol. 2024 Aug;45(6):1172–82.

11. Gould DS, Montenegro LM, Gaynor JW, Lacy SP, Ittenbach R, Stephens P, et al. A Comparison of On-Site and Off-Site Patent Ductus Arteriosus Ligation in Premature Infants. Pediatrics. 2003 Dec 1;112(6):1298–301.

12. Karamlou T, Diggs BS, Ungerleider RM, Welke KF. Evolution of treatment options and outcomes for hypoplastic left heart syndrome over an 18-year period. The Journal of Thoracic and Cardiovascular Surgery. 2010 Jan;139(1):119–27.

13. Kunde F, Thomas S, Sudhakar A, Kunjikutty R, Kumar RK, Vaidyanathan B. Prenatal diagnosis and planned peripartum care improve perinatal outcome of fetuses with transposition of the great arteries and intact ventricular septum in low‐resource settings. Ultrasound in Obstet &amp; Gyne. 2021 Sep;58(3):398–404.

14. Martini S, Galletti S, Kelsall W, Angeli E, Agulli M, Gargiulo GD, et al. Ductal ligation timing and neonatal outcomes: a 12-year bicentric comparison. Eur J Pediatr. 2021 Jul;180(7):2261–70.

15. Morris SA, Ethen MK, Penny DJ, Canfield MA, Minard CG, Fixler DE, et al. Prenatal Diagnosis, Birth Location, Surgical Center, and Neonatal Mortality in Infants With Hypoplastic Left Heart Syndrome. Circulation. 2014 Jan 21;129(3):285–92.

16. Patel M, Yu S, Romano JC, Bates K, Uzark K, Schumacher K, et al. Birth Location in Infants with Prenatally Diagnosed Hypoplastic Left Heart Syndrome. Pediatr Cardiol. 2022 Feb;43(2):301–7.

17. Purkey NJ, Ma C, Lee HC, Hintz SR, Shaw GM, McElhinney DB, et al. Distance from home to birth hospital, transfer, and mortality in neonates with hypoplastic left heart syndrome in California. Birth Defects Research. 2022 Jul 15;114(12):662–73.

18. Wong C, Mak M, Shivananda S, Yang J, Shah PS, Seidlitz W, et al. Outcomes of neonatal patent ductus arteriosus ligation in Canadian neonatal units with and without pediatric cardiac surgery programs. Journal of Pediatric Surgery. 2013 May;48(5):909–14.

19. WU J, LIU Y, SUN Y, ZHONG J, YU Y. Clinical study of inter-hospital transport of 237 neonates with acute and critical congenital heart disease. Chinese Journal of Applied Clinical Pediatrics [Internet]. 2020; Available from: https://pesquisa.bvsalud.org/portal/resource/pt/wpr-864243

20. Apfeld JC, Kastenberg ZJ, Sylvester KG, Lee HC. The Effect of Level of Care on Gastroschisis Outcomes. The Journal of Pediatrics. 2017 Nov;190:79-84.e1.

21. Du L, Pan WH, Cai W, Wang J, Wu YM, Shi CR. Delivery room surgery: an applicable therapeutic strategy for gastroschisis in developing countries. World J Pediatr. 2014 Feb;10(1):69–73.

22. Kitchanan S, Patole S, Muller R, Whitehall J. Neonatal outcome of gastroschisis and exomphalos: A 10‐year review. J Paediatrics Child Health. 2000 Oct;36(5):428–30.

23. Machaea SS, Chitnis MR, Nongena P. Prevalence of Gastroschisis and its Neonatal Mortality in the Eastern Cape Province tertiary Institutions. African Journal of Paediatric Surgery. 2023 Jan;20(1):46–50.

24. Muniz VM, Lima Netto A, Carvalho KS, Valle CSD, Salaroli LB, Zandonade E. Influence of birthplace on gastroschisis outcomes in a state in the southeastern region of Brazil. Jornal de Pediatria. 2021 Nov;97(6):670–5.

25. Murphy FL, Mazlan TA, Tarheen F, Corbally MT, Puri P. Gastroschisis and exomphalos in Ireland 1998–2004. Does antenatal diagnosis impact on outcome? Pediatr Surg Int. 2007 Oct 15;23(11):1059–63.

26. Singh SJ, Fraser A, Leditschke JF, Spence K, Kimble R, Dalby-Payne J, et al. Gastroschisis: determinants of neonatal outcome. Ped Surgery Int. 2003 Jun;19(4):260–5.

27. Vilela PC, Ramos De Amorim MM, Falbo GH, Santos LC. Risk factors for adverse outcome of newborns with gastroschisis in a Brazilian hospital. J Pediatr Surg. 2001 Apr;36(4):559–64.

28. Murthy K, Yanowitz TD, DiGeronimo R, Dykes FD, Zaniletti I, Sharma J, et al. Short-term outcomes for preterm infants with surgical necrotizing enterocolitis. J Perinatol. 2014 Oct;34(10):736–40.

29. Gibson A, Silva H, Bajaj M, McBride C, Choo K, Morrison S. No safe time window in malrotation and volvulus: A consecutive cohort study. J Paediatrics Child Health. 2024 Jun;60(6):206–11.

30. Jiang Y, Pan W, Wu W, Wang W, Sun S, Wang J. Can early surgery improve the outcome of patients with meconium peritonitis? A single-center experience over 16 years. BMC Pediatr. 2019 Dec;19(1):473.

31. Polites SF, Lautz TB, Jenkins TM, Dasgupta R. Implications of transfer status on bowel loss in children undergoing emergency surgery for malrotation. Journal of Pediatric Surgery. 2019 Sep;54(9):1848–53.

32. Zouari M, Ameur HB, Krichen E, Saad NB, Dhaou MB, Mhiri R. Risk factors for adverse outcomes following surgical repair of esophageal atresia. A retrospective cohort study. Diseases of the Esophagus. 2023 Mar 30;36(4):doac070.

33. Besendörfer M, Günster S, Linz K, Reutter HM, Diez S. Centralization as the key survival benefit in acute neonatal surgery. Front Pediatr. 2024 Mar 14;12:1382000.

34. Frassanito P, Serrao F, Gallini F, Bianchi F, Massimi L, Vento G, et al. Ventriculosubgaleal shunt and neuroendoscopic lavage: refining the treatment algorithm of neonatal post-hemorrhagic hydrocephalus. Childs Nerv Syst. 2021 Nov;37(11):3531–40.

35. Kancherla V, Ma C, Purkey NJ, Hintz SR, Lee HC, Grant G, et al. Factors Associated with Transfer Distance from Birth Hospital to Repair Hospital for First Surgical Repair among Infants with Myelomeningocele in California. Am J Perinatol. 2024 May;41(S 01):e1091–8.

36. Naicker D, Leola K, Mkhaliphi MM, Mpanza MN, Ouma J, Nakwa FL, et al. Single surgeon case series of myelomeningocele repairs in a developing world setting: Challenges and lessons. World Neurosurg X. 2023 Jul;19:100213.

37. Nelson CP, Bloom DA, Dunn RL, Wei JT. Bladder exstrophy in the newborn: A snapshot of contemporary practice patterns. Urology. 2005 Aug;66(2):411–5.

38. Parodi A, Giordano I, De Angelis L, Malova M, Calevo MG, Preiti D, et al. Post‐haemorrhagic hydrocephalus management: Delayed neonatal transport negatively affects outcome. Acta Paediatrica. 2021 Jan;110(1):168–70.

39. Ugwu R, Okoro P. Pattern, outcome and challenges of neonatal surgical cases in a tertiary teaching hospital. Afr J Paediatr Surg. 2013;10(3):226.
