# Supplementary material for: Association between transfer for surgery and mortality and disability among neonates in high income countries—A systematic review with meta-analysis
Source: PLoS One. 2025 Jul 31;20(7):e0327971. doi: 10.1371/journal.pone.0327971 (PMC12312895; doi:10.1371/journal.pone.0327971)
Supplement: S3 Table — (DOCX) [file pone.0327971.s005.docx]

**S3 Table: Definition of co-located care and surgical transfer**

| **Study ID** | **Definition of Co-located Care** | **Definition of Surgical transfer** |
| --- | --- | --- |
| **Congenital diaphragmatic hernia (CDH)** | | |
| Algert 2008 [20] | Co-located with a paediatric surgical unit, referred to as ‘co located’ | Transferred from other hospital |
| Al- Shanafey 2002 [21] | Patients born at study centre (IWK Grace health centre, Halifax) | Patients referred to study centre |
| Al Shareef 2024[22] | Born in the hospital (King Abdelaziz medical city, Jeddah) | Born outside the centre |
| Aly 2010[23] | Birth at surgical centre | Transferred to surgical centre |
| Boloker 2002[24] | Patients born in the surgical centre (Children’s hospital, New York) | Transferred cases to the surgical centre |
| Bryner 2009[25] | Not specified (NS) | NS |
| Carmichael 2020[26] | Infants not requiring transfer for CDH repair | Infants who require transfer from birth hospital to another hospital for CDH repair |
| Dahlheim 2003[27] | Neonates born in the ECMO center- University Children Hospital- Mannheim | Neonates born at other perinatal centres and transferred to University Children Hospital |
| Finer 1998[28] | Neonates born in the Royal Alexandra hospital | Neonates born at other perinatal centres and transferred to Royal Alexandra hospital |
| Gallot 2007 [29] | Born in the tertiary care hospital | Those born in peripheral hospitals and transferred to the tertiary centre |
| Goldshore 2023[30] | Children born in the pediatric hospital (Children Hospital of Philadelphia)): | Children who were delivered elsewhere |
| Khachane 2021 [31] | Infants born in the co-located perinatal unit at Childrens Hospital at Westmead, Sydney | Infants retrieved from outlying hospitals |
| Kim 2009[32] | NS | NS |
| Maldonado 2024[10] | Birth in a neonatal surgical unit | Designated tertiary neonatal unit for surgical conditions and transfer to a surgical centre (*tertiary designated*).  Tertiary neonatal unit not designated for surgical conditions and transfer to a surgical centre (*tertiary non-designated*).  Non-tertiary units without surgery and transfer to a surgical centre (*non-tertiary*). |
| Nagata 2013[33] | Born in the surgical centre | Those needing transfer to surgical centre |
| Nakayama 1985[34] | NS | NS |
| Nasr 2011[35] | Infant born in the same building as the NICU or in a different building connected by a tunnel or bridge to the NICU. | Infant born in a different building from the NICU, requiring transport.  Outborn may also include infants delivered in a hospital different from the one intended in which a prenatal diagnosis is made but delivery site plan is not met |
| Peiffer 2024[36] | Born in the Baylor College of Medicine | Born in non-health care facility, clinic and transfer from Oak Street Health |
| Reyes 1998[37] | Babies born at study centre [University of California, Irvine Medical Center ] | Transferred to study centre |
| Rocha 2008[38] | Infants born in São João NICU | Babies transferred to study centre |
| Sola 2010[39] | Surgery done in the birthing hospital | Those who required transfer for surgery |
| Stopenski 2021 [40] | Inborn was defined as neonates that were delivered and definitively treated at one of the participating CDHSG hospitals. | Outborn was defined as a neonate delivered in a separate institution and required transfer to a definitive care center. |
| Teo 2020[41] | Inborn were born within the hospital [KK Women’s and Children’s Hospital, Singapore] | Outborn were born outside and transferred to the hospital immediately post delivery |
| Wynn 2013[42] | Child born in the participating institution of DHREAMS study | Child transferred to the participating institution |
| **Congenital heart disease** | | |
| Bennett 2010[43] | Born at pediatric cardiac specialty care center | Born at any other hospital in Washington |
| Cave 2023[44] | Those born in the tertiary care centre (Yorkshire, United Kingdom) | Those who required transfer to tertiary care centre for surgery |
| Cloete 2018[45] | Antenatally diagnosed cases born in the cardiac convention centre | Those who were transferred to the surgical centre within 48 hours of birth |
| Garne 2007[46] | Paediatric cardiology centre | Referred cases from other hospital and home delivery |
| Hamzah 2020[47] | Those born in the surgical facility | Those who were transferred in for surgery |
| Mattia 2024[48] | Cardiac intervention team from Phoenix children hospital would perform BAS at bedside for prenatally diagnosed d-TGA delivered at a designated center 2.1miles away | Prenatally diagnosed d-TGA transferred from delivery center to Phoenix childrens for BAS |
| Purkey 2021[49] | Those who were born into the same hospital as the intervention | Transferred from the birth hospital to the intervention centre after birth |
| Swartz 2017[50] | Those born at the cardiac surgery center (The University of Rochester Medical Center, New York) | Those who were transferred for their surgical procedure |
| Thomas 2023[51] | Those born at University of Michigan (U of M) | Those who were transferred to U of M for surgical procedure |
| Veal 2019[52] | Born in tertiary maternity unit adjacent to specialist paediatric ICU | Those born in district general hospital |
| **Esophageal atresia, Tracheo-esophageal fistula** | | |
| Schlee 2022[70] | Patients who were born in study institution (University Hospital Goethe University Frankfurt) | Those postnatally referred to study institution |
| Sfeir 2021[71] | NS | NS |
| Wang 2014[72] | Those born in the surgical centre | Interhospital transfer to the surgical centre |
| **Gastroschisis** | | |
| Abdel-Latif 2008[57] | NS | NS |
| Clark 2010[58] | NS | NS |
| Dalton 2016[59] | Those who were delivered within study center (Children's Mercy Hospital Kansas City) | Those delivered elsewhere who were subsequently transferred for pediatric surgical care |
| Hong 2018[60] | NS | Outborn status was defined by delivery outside of reporting center and admission to reporting center within 28 days of birth without prior discharge home. |
| Lee 2024[62] | Infants born at tertiary surgical NICU at King’s College Hospital, UK | Born outside of King’s college hospital and transferred by specialist neonatal transfer teams |
| Kandasamy 2010[61] | Those born at study centre | Those transferred to study centre for surgical repair |
| Nasr 2012[63] | Born in the same building as the NICU, or in a different building connected by a tunnel or bridge to the NICU | -Born in a different building from the NICU, requiring a trip in an ambulance or other form of transportation.  -Delivery in a hospital different from the one intended-prenatal diagnosis made |
| Quirk 1996[64] | Delivered at the University Hospital of Arkansas with immediate transfer to Arkansas Children's Hospital for surgical repair | Infants transferred to Arkansas Children's Hospital after delivery at an outlying hospital |
| Rinehart 1999[65] | Delivered at the University of Mississippi Medical Center | Transferred to the University of Mississippi Medical Center |
| Savoie 2014[66] | Patient being born at the hospital where their surgical repair occurred | All patients not designated as inborn were Outborn |
| Stoodley,1993[67] | At Bristol Maternity Hospital | Transfer to study centre |
| Stringer, 1991[68] | Born at Hospital for sick children or University college hospital, London | Transferred postnatally to Hospital for sick children or University college hospital, London |
| Youssef 2016[69] | University free-standing children's  hospitals and medical centres with established pediatric surgical services. | Transferred postnatally to medical centres with established pediatric surgical services. |
| **Meconium Peritonitis, Intestinal atresia** | | |
| Chen 2019[73] | Born in Mackay memorial hospital | Neonates who were transferred to Mackay memorial hospital |
| Erickson 2016[74] | Born in study hospital | Transferred from another hospital after delivery |
| Paradiso 2011[75] | Those delivered in the institution (San Camillo-Forlanini Hospital) | All those who were referred to the institution |
| Wong 2023[76] | Born in study hospital (Queen Mary hospital, Hong Kong) | Transferred from another hospital after delivery |
| **Necrotising enterocolitis and spontaneous intestinal perforation** | | |
| Fullerton 2016[53] | NS | NS |
| Granger 2023[7] | Infants with NEC/Focal intestinal perforation (FIP) presented to surgical unit | Infants transferred to surgical unit |
| Kelley Quon 2012[54] | Born at study centre (level 2A-3C) | Transfer- to surgical NICU 3B or 3C (99% of surgeries occurred in these centres) |
| Loh 2001[55] | Infants cared for in centres with on-site surgical facilities (group 1) | Infants transferred from centres without surgical facilities |
| Zamrik 2018[56] | The Children’s Hospital Oberhausen **(center 2)** comprising an on-site pediatric surgery department | Infants transferred from Children’s Hospital Wuppertal **(center 1) that** had a 40km distant pediatric surgical facility |
| **Others** | | |
| Kancherla 2021[77] | Those operated in birth hospital | Transferred from different hospital |
| Yang 2015[78] | Those operated in birth hospital | Transferred from different hospital |

CDH: Congenital diaphragmatic hernia; CDHSG: CDH study group; NS: Not specified; CHOP: Children’s hospital of Philadelphia; FIP: Focal intestinal perforation; NICU: Neonatal intensive care unit; DHREAMS: Diaphragmatic Hernia Research and Exploration- Advancing Molecular Science; NEC: Necrotizing enterocolitis; BAS: Balloon Atrial Septostomy; TGA: Transposition of Great Arteries
