# Supplementary material for: Association between transfer for surgery and mortality and disability among neonates in high income countries—A systematic review with meta-analysis
Source: PLoS One. 2025 Jul 31;20(7):e0327971. doi: 10.1371/journal.pone.0327971 (PMC12312895; doi:10.1371/journal.pone.0327971)
Supplement: S4 Table — (DOCX) [file pone.0327971.s006.docx]

**Outcomes described in the systematic review (not meta-analyzed)**

**Mortality**

| **Surgical condition** | **Outcome** | **Study** | **Odds ratio 95% (CI)** |
| --- | --- | --- | --- |
| Gastric perforation | Crude Mortality | Yang 2015 | 7.36 [0.31, 176.41] |
| Neural tube defects | Crude Mortality | Algert 2008 | 4.00 [0.31, 51.03] |
| Trachea -esophageal fistula | Risk adjusted Mortality | Sfeir 2021 | 0.97 [0.39, 2.39] |
| Neural tube defects | Risk adjusted Mortality | Kancherla 2021 | 0.31 [0.11, 0.87] |

**Neurodevelopment**

| **Surgical condition** | **Outcome** | **Study** | **Odds ratio 95% (CI)** |
| --- | --- | --- | --- |
| Necrotizing enterocolitis | Neurodevelopmental disability at 2 years | Granger 2022 | 0.65 [0.20, 2.10] |
